# A systematic review of artificial intelligence in radiotherapy associated cardiovascular toxicity

**DOI:** 10.1186/s40959-026-00528-5

**Published:** 2026-06-19

**Authors:** Vivian Salama, Brandon M. Godinich, Nathaniel Dunham, Troy Nguyen, Sijin Wen, Joseph A. Schmidlen, Wesley Cox, Peyton M. Lilly, Jeffrey Ryckman, Ramon Alfredo Siochi, Ashkan Emadi, Christopher M. Bianco, George G. Sokos, Raymond R. Raylman, David A. Clump, Mina F. Hanna, Phillip M. Pifer

**Affiliations:** 1 Department of Radiation Oncology, West Virginia University, School of Medicine, WVU Cancer Institute, Morgantown, WV USA; 2 Department of Medical Education, Paul L. Foster School of Medicine, Texas Tech Health Sciences Center, El Paso, TX 79430 USA; 3 Department of Radiology, West Virginia University, School of Medicine, Morgantown, WV USA; 4 Department of Epidemiology and Biostatistics, West Virginia University, School of Public Heath, Morgantown, WV USA; 5 West Virginia School of Osteopathic Medicine/Iredell Health System, Lewisburg, WV USA; 6 Department of Medical Oncology, West Virginia University, School of Medicine, WVU Cancer Institute, Morgantown, WV USA; 7 Department of Cardiology and Cardiac Surgery, West Virginia University, School of Medicine, WVU Heart and Vascular Institute, Morgantown, WV USA; 8 Department of Medical Oncology and Radiation Oncology, West Virginia University, P.O. Box 9162, 1838 Cancer Center, Morgantown, WV 26506-9300 USA

**Keywords:** Risk prediction, Cardio-oncology, Machine learning, Treatment planning, Cancer survivorship, Deep learning, Radiation therapy, Cardiovascular imaging, Cardiac contouring

## Abstract

**Background:**

Cardiovascular toxicity (CVT) is a major concern after radiotherapy (RT), contributing to morbidity and mortality among cancer survivors. Artificial intelligence (AI) may improve risk prediction and RT planning; however, its role in RT-associated CVT remains unclear. This study aimed to systematically evaluate AI applications and study quality in this field.

**Methods:**

A PRISMA-guided systematic review of PubMed, Ovid EMBASE, Cochrane Library, and Web of Science was conducted through October 1, 2025. Eligible studies included original human research in English applying AI to CVT or imaging in cancer populations receiving RT. Predictive and imaging studies were evaluated using TRIPOD + AI/PROBAST and CLAIM/QUADAS-2, respectively. Exploratory meta-analysis of performance metrics was conducted where feasible.

**Results:**

Sixty-five studies were included, comprising AI prediction models (*n* = 31, 48%) and cardiovascular imaging applications (*n* = 34, 52%). Deep learning was the most common approach (45/65, 69%) and demonstrated the highest predictive performance (median AUC = 0.82; median sensitivity = 0.83). Calibration assessment (3/31, 10%) and external validation (6/31, 19%) were limited. Meta-analysis demonstrated an overall predictive model accuracy of 0.83 (95% CI: 0.77–0.87). Imaging models performed well for larger cardiac structures (overall median DSC = 0.85, range: 0.76–0.94), while coronary artery segmentation remained challenging. Average TRIPOD + AI and CLAIM adherence were 79% and 71%, respectively. Most predictive (97%) and imaging (82%) studies were rated at high risk-of-bias.

**Conclusion:**

AI shows promise for RT-associated CVT prediction and imaging but is underdeveloped for routine clinical implementation. Heterogeneity, limited validation, and methodological limitations highlight the need for standardized endpoints, external validation, and prospective clinical evaluation.

**Supplementary Information:**

The online version contains supplementary material available at https://doi.org/10.1186/s40959-026-00528-5.

## Highlights

- Radiation therapy increases long-term cardiovascular toxicity in cancer survivors.
- AI shows promise for prediction and imaging but lacks robust validation.
- High reported AI performance is limited by heterogeneity and incomplete reporting.
- Methodological quality remains limited despite TRIPOD-AI and CLAIM guidance.
- Standardized endpoints, external validation, and prospective clinical integration are urgently needed.

**Supplementary Information:**

The online version contains supplementary material available at https://doi.org/10.1186/s40959-026-00528-5.

## Introduction/background

**Fig. 1:**
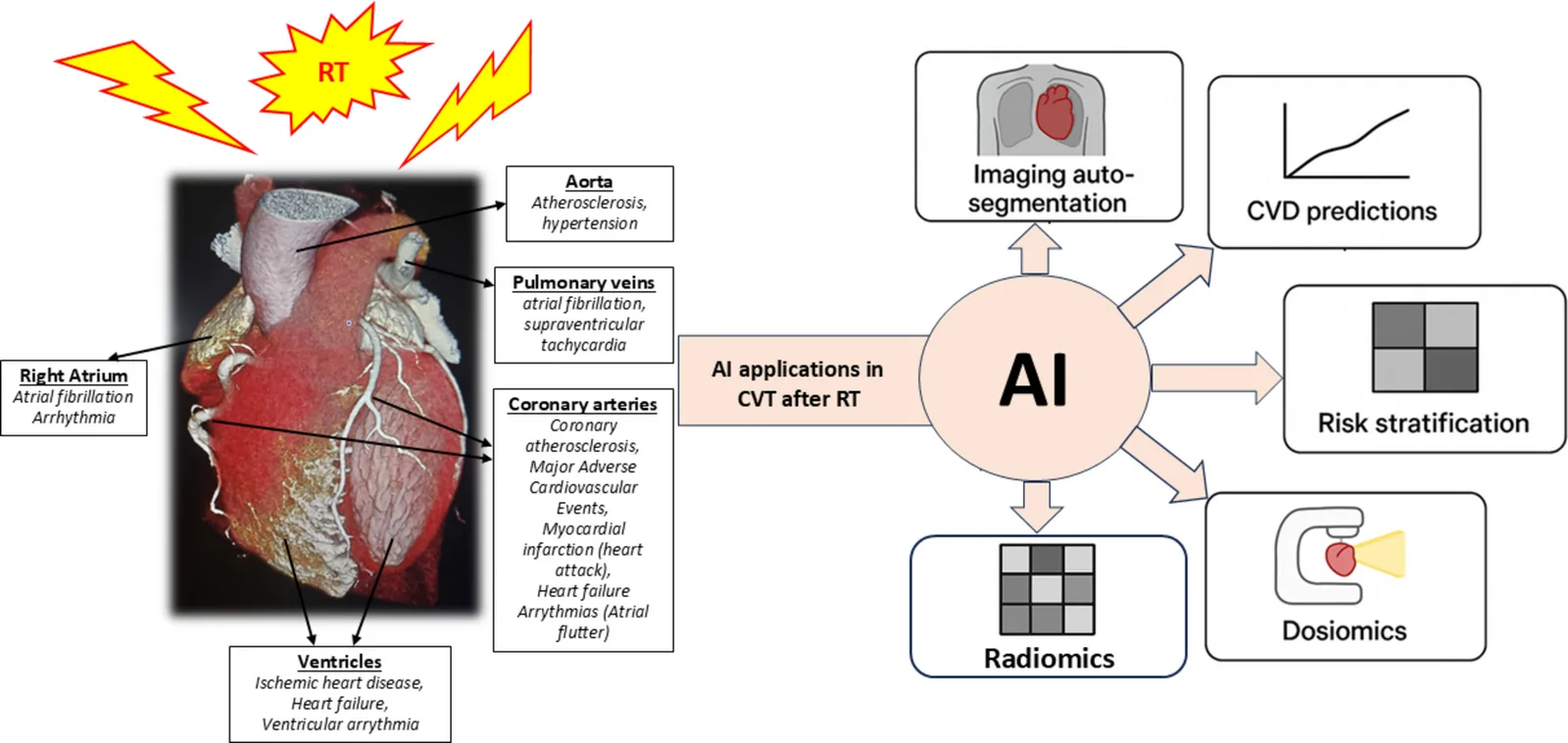
Illustration demonstrates post-radiation cardiovascular toxicity (CVT) and the artificial intelligence (AI) applications in the field of CVT after radiation therapy (RT)

Cancer therapies can damage the cardiovascular system, resulting in cardiovascular toxicities (CVT) that negatively impact outcomes and survival [1]. Radiation therapy (RT) is a cornerstone modality of cancer treatment, and has significantly improved survival outcomes across multiple malignancies, including breast, lung, and thoracic cancers [2–4]. However, exposure of cardiac and vascular structures to ionizing radiation can result in a spectrum of cardiovascular complications ranging from subclinical myocardial injury to clinically significant conditions including ischemic heart disease, heart failure, arrhythmia, valvular disease, and pericardial disease (Fig. 1) [5–7]. These cardiovascular complications have become an increasingly important concern in cardio-oncology because they negatively affect quality of life (QoL), survivorship outcomes, and overall survival.

Radiation-associated cardiovascular injury is influenced by multiple interacting factors including patient characteristics, baseline cardiovascular risk, treatment parameters, and biological responses to therapy. Reported rates of RT-associated CVT vary across populations and treatment settings, with approximately 20–30% of patients with lung or esophageal cancers developing CVT after RT [8, 9]. Substantially higher rates (55%) have been reported among patients receiving mediastinal radiation for lymphoma [10]. As cancer survival improves, cardiovascular disease has emerged as a leading cause of long-term non-cancer morbidity and mortality among survivors [11–13]. Early identification of patients at increased risk of CVT therefore remains an important challenge in cardio-oncology.

Artificial intelligence (AI) and machine learning (ML) methods have increasingly been incorporated into oncology research and clinical workflows because of their ability to analyze large and complex datasets [14–16]. In radiation oncology, AI has demonstrated utility in treatment planning, auto-segmentation, outcome prediction, and personalized decision support [17–20]. Different AI applications could be beneficial in the field of post-treatment CVT, as illustrated in Fig. 1. Chen et al. [21], Loap et al. [22] and Borges et al. [23] showed the efficacy of deep learning (DL) in imaging auto-segmentation of the cardiovascular structures for better RT planning to reduce the incidence and severity of post-radiation CVT. Zhou et al. [24], Qiao et al. [25], Bentriou et al. [26] and Dincer et al. [27] used ML classification and regression models for CVT prediction after RT.

Despite the rapid expansion of AI in cardio-oncology and radiation oncology, its application to CVT in populations receiving RT remains largely unappreciated. Existing studies vary widely in methodology, data sources, outcome definitions, validation strategies, and reporting quality. While individual studies explored AI-based prediction models, cardiac imaging applications, and treatment planning tools, a comprehensive evaluation of the current evidence, methodological quality, and existing limitations is lacking. Understanding the current landscape is critical for clinicians, researchers, and computational scientists aiming to improve survivorship outcomes and QoL of patients.

Our scientific questions in this review were:

1. What are the key applications of AI models for identifying and evaluating CVT in patients receiving cancer treatment with RT as a component of care?
2. Which AI tools are commonly used to address/predict treatment-associated CVT in populations receiving RT?
3. What is the quality of studies conducted in AI in cardio-oncology among patients undergoing treatment that includes RT
4. To what degree do the existing AI models in cardio-oncology follow the AI reporting and transparency guidelines?

This systematic review aimed to evaluate and summarize the existing literature on AI applications related to CVT among patients undergoing treatment that includes RT. Specifically, AI methodologies, model performance, reporting quality, and risk-of-bias. Current limitations and future opportunities for clinical translation were also identified.

## Materials and methods

### Protocol registration

Registration of this review was conducted in the international prospective register database of systematic reviews (PROSPERO) on September 29th, 2025 (ID number: CRD420251158012) in the context of human health care.

### Search strategy

A comprehensive systematic search of PubMed, Ovid EMBASE, Cochrane Library, MEDLINE, and Web of Science databases was conducted for publications of original human research in English up to October 1, 2025. The concepts searched included: “artificial intelligence”, “machine learning”, “deep learning”, “neural networks”, “cardiovascular toxicity”, “cardiovascular diseases”, “radiotherapy”, “cardio-oncology”. A Combination of the concepts/terms was done using OR/AND Boolean operators. The search strategy using Boolean operators was described in Supplementary File S1.

### Screening process

Articles identified from databases were uploaded into “Rayyan,” an AI-powered systematic review management platform for screening. Duplicated articles were identified and removed through Rayyan. The screening process was blindly conducted with three independent reviewers (BG, ND, and PL), then a fourth reviewer (VS) solved the conflicts. Screening of the titles and abstracts was done first, and then screening of the full text was done for the reports for retrieval. The primary outcome of this review was to identify the applications and quality of AI approaches in either predicting, preventing, or managing treatment-associated cardiovascular toxicity in patients with cancer receiving care that includes RT.

#### Inclusion criteria

Included studies had to meet all the following criteria: (1) full original research, (2) Available in English, (3) involved human subjects, (4) studies applied AI or ML models, (5) cardiovascular related outcome (including cardiovascular diseases or cardiovascular imaging), (6) studies in the cancer population received radiation therapy or chemo-radiotherapy, and (7) models were tested for performance.

#### Exclusion criteria

Articles were excluded if they met any of the following criteria: (1) irrelevant or out of scope study, (2) not a full original study (e.g., conference abstract, review articles, letter, or editorial), (3) non-human study (e.g., animal or cell lines), (4) no AI or ML model was applied, (5) no cardiovascular related outcome, or (6) no radiation therapy received.

### Data synthesis, extraction, collection, and analysis

This review followed the Preferred Reporting Items for Systematic Reviews and Meta-Analysis (PRISMA) guidelines (PRISMA checklist in Supplementary File S2). Data from final included articles were extracted into Excel sheets. Extracted data included study characteristics and demographics (author, year, and journal), study design, number of population/participants, cancer type, AI method/model, input and outcome variables, and AI model performance metrics (e.g., AUC, accuracy, sensitivity, specificity and others). All included studies were categorized into two main groups based on the main applications of AI: (1) studies applied AI in cardiovascular toxicity prediction and stratification in patients receiving cancer treatment with RT as a component of care (2) studies applied AI models in cardiovascular imaging analysis in patients receiving RT. Due to significant heterogeneity in study design, sample sizes, AI models, outcomes, and reported performance metrics, a comprehensive pooled meta-analysis across all studies was not fully performed. However, exploratory meta-analysis was conducted for selected performance measures (accuracy) where sufficient comparable data were available. Classification accuracy was used as the primary effect measure for predictive models. Studies were considered eligible for quantitative synthesis if they reported comparable performance metrics and sufficient summary statistics for effect estimation. Individual study estimates, number of participants, and corresponding 95% confidence intervals were extracted directly from the original publications when available. Pooled estimates were calculated using both fixed-effects and random-effects models. When necessary, reported summary statistics were converted to a common format to facilitate synthesis. Results were visually summarized using forest plots. Summary statistics including median, interquartile range (IQR), and range were calculated for reported model performance metrics using JMP.Pro Edition 18.2.1 software. Meta-analysis was conducted using R studio.

### Results’ presentation

Results were summarized in a tabular format to summarize study characteristics, AI methodologies, and conclusions. Visualizations (e.g. bar charts, pie charts, flow diagrams and forest plots) were generated using GraphPad prism to illustrate the trend of publication, cancer types, type of research study, TRIPOD + AI and CLAIM adherence, and risk-of-bias assessment.

### Data quality and risk-of-bias assessment

Three reviewers (BG, ND, and PL) conducted blind screening of the identified articles. Three reviewers (BG, ND, and PL) assessed full texts of the included articles thoroughly. A fourth reviewer (VS) resolved conflicts. The materials and methods of the studies and the results sections were assessed. Non-full articles, or full articles that could not be accessed were excluded.

To evaluate the overall quality of the included articles, a rigorous assessment was conducted, focusing on evaluation of both risk-of-bias and adherence to AI reporting guidelines for each individual article. This was based on the application of AI and the type of the study. For studies applying AI or ML for predictive tasks and risk stratification, the risk-of-bias was assessed using the Prediction Model Risk of Bias Assessment Tool (PROBAST), which examines four domains (participants, predictors, outcomes, and analysis) to determine the overall risk-of-bias [15, 16, 28, 29]. Adherence to the updated AI-Transparent Reporting of a Multivariable Prediction Model for Individual Prognosis or Diagnosis (TRIPOD + AI) guidelines was analyzed using the TRIPOD + AI checklist. This checklist covers 27 items including overall 51 questions and subitems specified for AI models [16, 30–32]. For studies/articles applying AI in cardiovascular imaging analysis, an updated Checklist for Artificial Intelligence in Medical Imaging (CLAIM) [33] tool was used to assess the quality and consistent reporting. Risk-of-bias for the included imaging-AI studies was evaluated using the Quality assessment of diagnostic accuracy studies (QUADAS-2) tool across 11 questions covering 4 domains: patient selection, index test, reference standard, flow, and timing [34, 35]. If any answer for any question in the domain was assessed as “*No*”, the final risk-of-bias of the domain was judged as high.

Formal assessment of publication bias and certainty was not fully performed due to the heterogeneity of study designs. However, selective reporting within studies was indirectly evaluated through adherence to reporting guidelines (TRIPOD + AI and CLAIM), which assess transparency in outcome reporting and model performance metrics. Additionally, the overall certainty of the evidence was qualitatively assessed by considering the risk of bias identified through the PROBAST and QUADAS-2 evaluations.

## Results

### Search and screening results

**Fig. 2:**
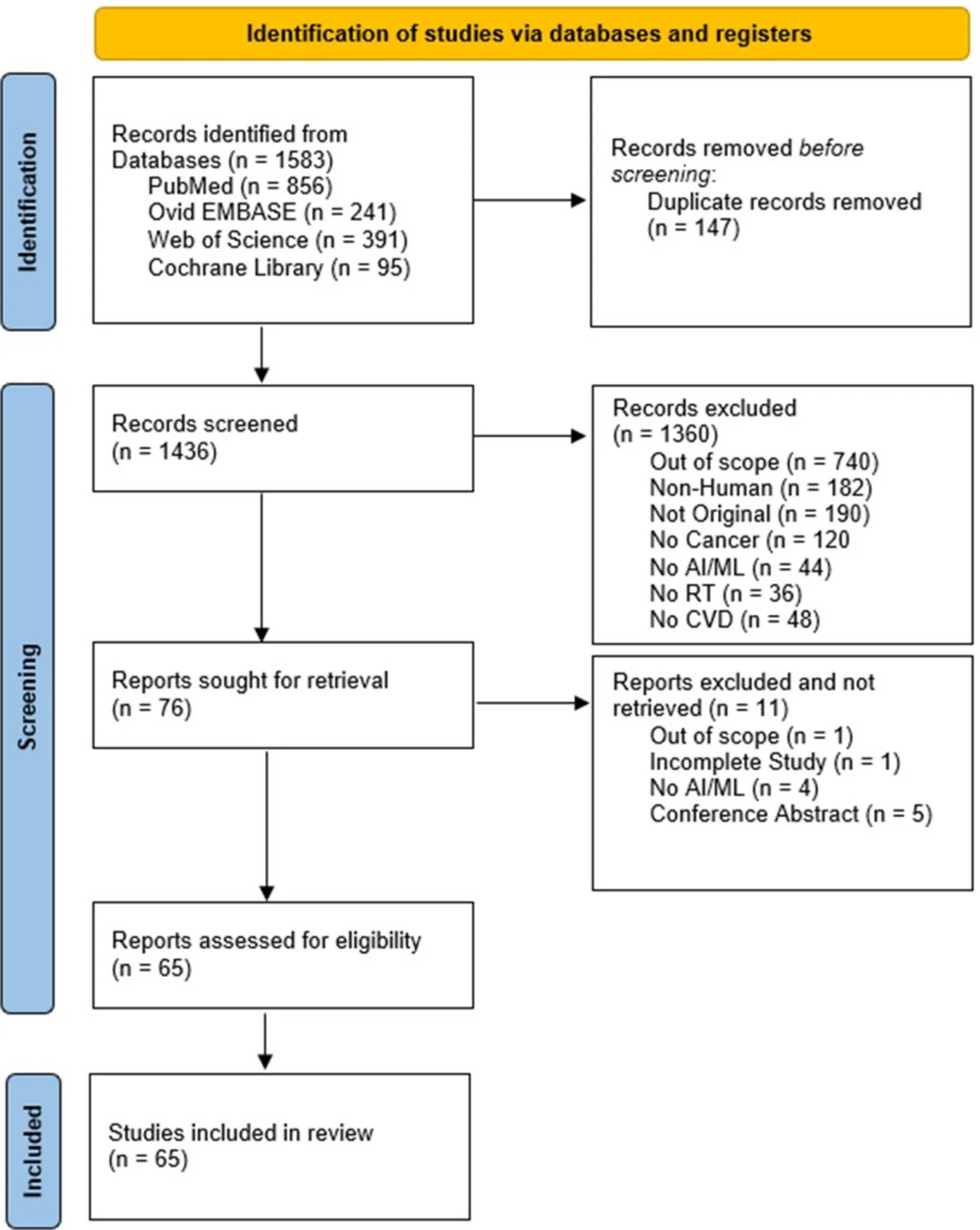
PRISMA flow diagram for systematic review of artificial intelligence in cardiovascular toxicity after radiation therapy

Our comprehensive database search resulted in a total of 1583 studies from PubMed (*n* = 856), Ovid EMBASE (*n* = 241), Cochrane Library (*n* = 95) and Web of Science (*n* = 391). A total of 65 articles met all inclusion criteria. The full search process was illustrated in the PRISMA flow chart (Fig. 2).

### Trend, study design, cancer type and participants

**Fig. 3:**
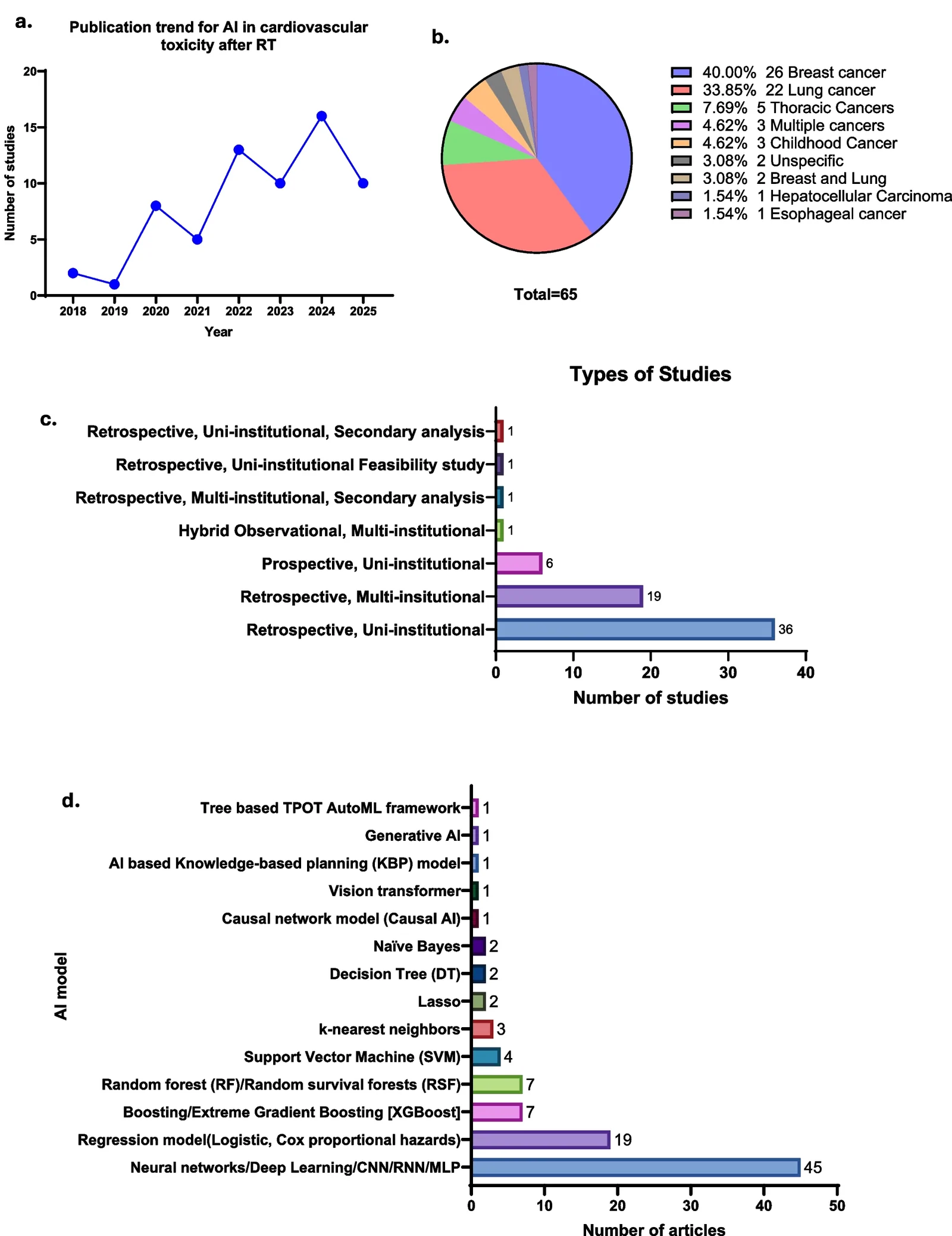
Publication characteristics of included articles.** a** Publications trend for AI models applied in cardiovascular toxicity prediction or CV imaging analysis in patients with cancers who received RT. **b** Frequency of the types of cancers studied in included articles. **c** Types and numbers of studies across the included articles of this review. **d** Types of AI or ML models and the number of studies investigated each model

All included studies were published from 2018 to 2025, with an increase in the number of publications from 2 per year in 2018 to 16 and 10 per year in 2024 and 2025, respectively (Fig. 3a). The most common type of cancer included in these studies was breast cancer (*n* = 26, 39%) followed by lung cancer (*n* = 22, 34%) (Fig. 3b). The literature was dominated by single-institution, retrospective studies (*n* = 36, 55% studies) (Fig. 3c). The median sample size to build the models was 177 (IQR 54–984), while 2 articles did not specify sample size. One study was a notable outlier with an exceptionally large cohort size (*N* = 148,755,036). Several types of AI models were investigated (Fig. 3d) with neural networks (deep learning) being the most used approach (*n* = 45, 69%), especially in imaging-based analysis. This was followed by the regression models (*n* = 19, 29%), then classification ensemble models (e.g., Boosting models and random forest) (*n* = 7, 11% per model).

### Study characteristics and AI applications

The characteristics and demographics of the 65 included studies were illustrated in Supplementary Table 1. Out of the 65 included studies, 34 (52%) applied AI for imaging analysis, while 31 (48%) applied AI or ML for prediction, risk stratification, and patient selection of CVT following RT.

#### Artificial Intelligence models applications in cardiovascular toxicity prediction and stratification in patients receiving cancer treatment with RT as a component of care

##### Models’ characteristics and endpoints

**Fig. 4:**
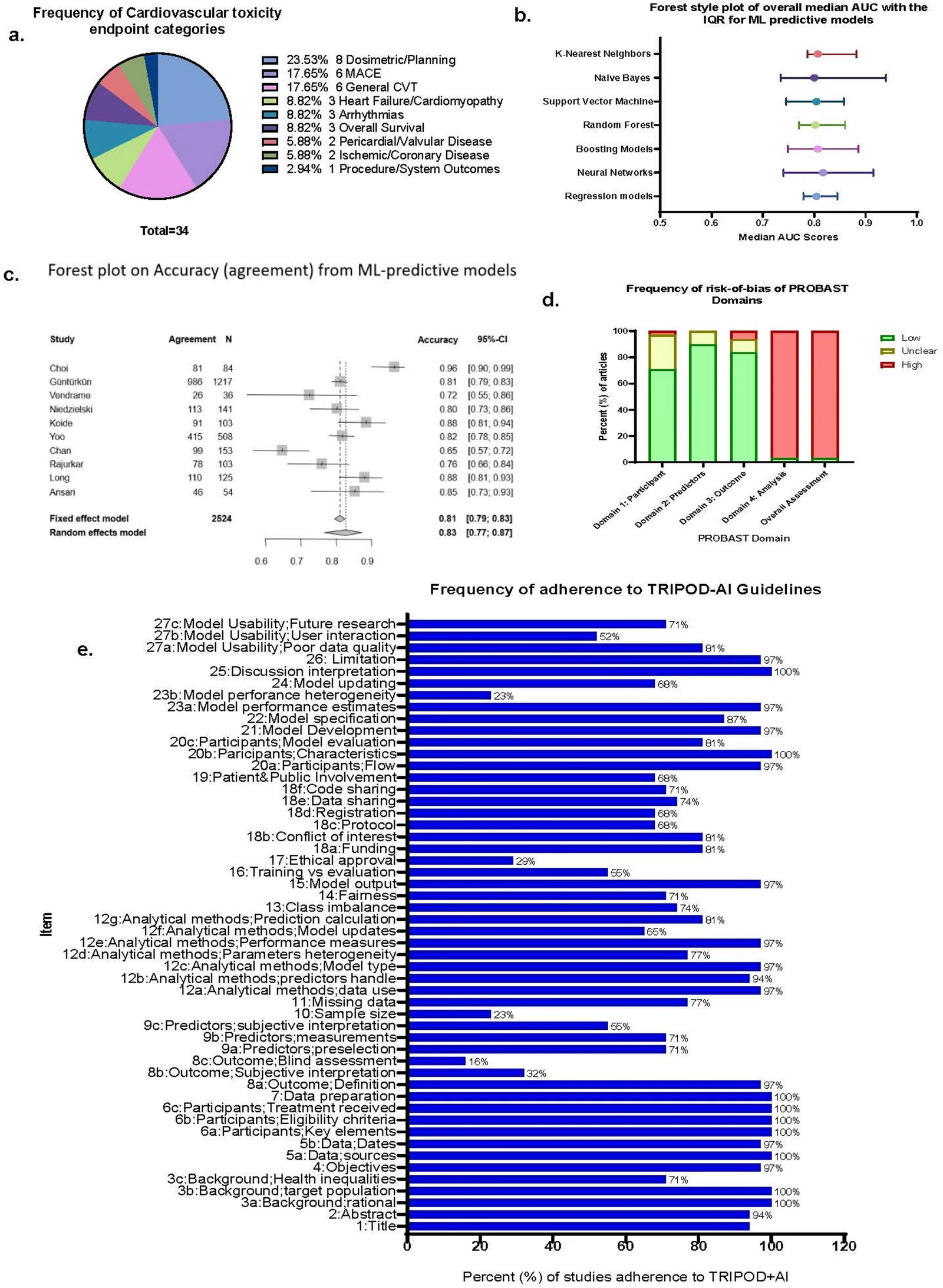
Characteristics, Endpoints, performance, Quality and Risk-of bias analysis of included studies group applied AI in cardiovascular toxicity prediction and risk stratification in populations receiving RT. **a** Pie chart of the frequency of cardiovascular toxicity endpoint categories. **b** Median ROC-AUC and the Inter-Quantile Range (IQR) of AI models across the included predictive AI modeling studies. **c** Forest plot summarizing the reported classification average accuracy of machine learning predictive models. Horizontal lines representing corresponding 95% confidence intervals (CI). Diamonds indicate pooled estimates using fixed-effects and random-effects models. **d** Frequency of risk-of-bias assessment of included studies applied predictive AI or ML models for cardiovascular toxicity prediction and risk stratification in populations receiving RT, using the Prediction Model Risk-of-Bias Assessment Tool (PROBAST). **e** Frequency of adherence of studies applied predictive AI or ML models for cardiovascular toxicity prediction and risk stratification in populations receiving RT, to the AI specific Transparent Reporting of a multivariable prediction Model for Individual Prognosis or Diagnosis (TRIPOD + AI) guidelines

Across the 31 predictive modeling studies, considerable variability existed in endpoint selection and outcome definitions (Fig. 4a). Major adverse cardiac events (MACEs) were the most frequently used clinical endpoint, reported in 6/31 (19%) predictive modeling studies. MACEs were defined as composite cardiovascular outcomes including myocardial infarction, coronary artery disease, heart failure, or stroke. They were used with grading commonly based on Common Terminology Criteria for Adverse Events (CTCAE) ≥ 3 or clinically judged cardiovascular events. General CVT endpoints were used in 6/31 (19%) studies including treatment-related cardiotoxicity without restriction to a single diagnosis. Less commonly studied outcomes included heart failure/cardiomyopathy, arrhythmias, pericardial or valvular disease, ischemic heart disease, procedure-based outcomes, and overall survival, each reported in only 1–3 studies. A significant proportion of studies (8/31, 26%) used dosimetric or treatment-planning endpoints rather than clinical cardiac events. These included prediction of mean heart dose or cardiac substructure dose.

##### Models’ validation and evaluation

Model validation mostly relied on internal validation strategies, with cross-validation used in 12/31 (38%) studies followed by independent hold-out testing in 9/31 (29%) studies. External validation using separate patient cohorts was performed in only 6/31 (19%) studies, reflecting the practical and methodological challenges of external validation in this setting. Several studies incorporated model comparison or benchmarking against conventional dosimetric or radiomics approaches, showing improved performance of ML dosiomics-based methods over traditional dose-volume metrics. Few studies (4/31, 13%) utilized interpretability-driven validation through explainable AI techniques to confirm meaningful cardiac substructure contributions. Only one study validated causal inference outputs by comparison with established medical literature and expert clinical review rather than predictive accuracy metrics Bernasconi et al. [36].

For evaluation of the performance of ML predictive models, most studies used discrimination metrics for classification/predictive models [e.g., Receiver Operating-Area Under the Curve (AUC), sensitivity/recall, specificity/precision, and accuracy]. Twelve out of the 31 (39%) studies did not report any of the discrimination metrics, raising concerns regarding reproducibility of model performance. Out of these 31 studies, 16 (52%) reported the model’s AUC. Supplementary Fig. 1 demonstrates average AUC with 95% confidence intervals (CI) of available data and studies. Median AUC scores with the range and IQR were summarized in Supplementary Table 2. Approximately half of studies reported AUC values, with median AUCs clustering around or above 0.80 (Fig. 4b). Neural network models demonstrated the highest discriminatory performance (median AUC = 0.82; IQR: 0.74–0.92; range: 0.69–0.98), although with notable variability. This was followed by K-nearest neighbors and boosting models, both with median AUCs of 0.81.

In terms of other discrimination metrics, 22/31 (71%) did not report the ML models’ sensitivity or specificity, and 21/31 (68%) did not report the model’s accuracy. Median sensitivity scores and specificity scores were summarized in Supplementary Table 3 and 4, respectively. Neural network models demonstrated the highest performance, achieving a median sensitivity of 0.83 (IQR: 0.82–0.85; range: 0.80–0.86), followed by regression-based models, (median sensitivity:0.82; IQR: 0.73–0.90; range: 0.66–0.96). Only one article reported the F1-Score of the AI predictive model Vendrame et al. [37]. Random forest models demonstrated the highest specificity (median = 0.83; IQR: 0.79–0.86), followed by boosting models (specificity = 0.81) derived from a single study. Neural network models showed a median specificity of 0.80 with broader dispersion (IQR: 0.72–0.88). Regression-based models demonstrated slightly lower specificity (median specificity = 0.78; IQR: 0.69–0.86). Overall, tree-based ensemble methods, particularly random forests, showed the most favorable and stable specificity, whereas neural networks and regression approaches exhibited greater variability across studies.

Calibration was notably limited across studies (3/31, 10%) despite its importance in comparing predicted and observed risk estimates. Niedzielski et al. [38] used the calibration curve and tested the calibration slope/intercept for the Elastic net logistic regression model: 1.356 / 0.235 (cross-validated heart + substructures model); 1.352 / 0.174 (final fitted model) [38]. Similarly, Tohidinezhad et al. [39], tested the calibration of the regression model using the calibration curve which showed good conformation of the predicted probabilities versus the actual outcomes for probabilities ranging between 0.1 and 0.9. Lee et al. assessed the multivariable logistic regression–based normal tissue complication probability (NTCP) model using he Hosmer–Lemeshow (H–L) goodness-of-fit test. The model demonstrated good calibration in both the development cohort (*P* = 0.565) and the validation cohort (*P* = 0.800) [40].

##### Exploratory meta-analysis of model performance (accuracy)

To provide an additional quantitative overview of predictive model performance across studies, exploratory pooled analyses of selected comparable metrics (AUC, sensitivity, and specificity) were performed and presented in Fig. 4c. and Supplementary Fig. 2. The pooled random-effects estimate for accuracy demonstrated an overall accuracy of 0.83 (95% CI: 0.77–0.87), while the fixed-effects model demonstrated an overall accuracy of 0.81 (95% CI: 0.79–0.83). However, substantial heterogeneity across study populations, sample sizes, cardiovascular endpoints, AI methodologies, and reporting performance metrics should be considered when interpreting these findings. Since Most studies did not report the AUC variability (e.g., 95% CI), developing actual meta-analysis of AUC performance for all studies was not feasible.

##### Quality of studies

The TRIPOD + AI checklist overall adherence averaged 79% (SD = 22.68%, 95%CI = 72.36–84.99) across all items. Core scientific reporting domains, such as title, abstract, objectives, data sources, outcome definitions, participant characteristics, model development, performance reporting, and discussion demonstrated high adherence (≥ 90–100%). Critical gaps included ethical approval (29%), blinded outcome assessment (16%), sample size justification (23%), handling of performance heterogeneity (23%), and model updating (65%). Open science practices including code and protocol sharing were also low (68–71%). Model usability was rarely addressed (52%) (Fig. 4e).

##### Risk-of-bias assessment

Using the PROBAST checklist, 30/31(97%) studies were rated as having a high overall risk-of-bias, driven entirely by the analysis domain (Domain 4), where 30/31 (97%) scored high risk due to inadequate handling of overfitting, weak validation, and incomplete performance reporting. Predictors (Domain 2) and outcomes (Domain 3) fared better with 90% and 84% rated as low risk, respectively. Participant selection (Domain 1) was adequate in 71% of studies. The near-universal failure in the analysis domain means that even studies with strong predictor and outcome reporting lack the analytical rigor needed for clinical application (Fig. 4d). Detailed risk-of-bias per study was included in Supplementary File S3.

#### Artificial Intelligence models applications in cardiovascular imaging in patients with cancer who received RT as a component of care

##### Models’ characteristics

Across the 34 included studies, DL approaches were most predominant (30/34, 88%). Most of these DL models were convolutional neural network (CNN)-based models, primarily U-Net variants.

Across imaging studies, AI applications in cardiovascular imaging for radiation treatment were clustered into three dominant categories: 1) Cardiac or cardiopulmonary substructures segmentation was the most common application (22/34, 65%). 2) Coronary or thoracic aorta calcification quantification (8/34, 24%). 3) Functional radiomics or dosiomics constituted (4/24, 11%). Among the segmentation-focused studies, CNN-based auto-segmentation models were used in more than 85%. These models were primarily developed to automate whole-heart and cardiac substructure contouring on radiotherapy planning CT, aiming to improve radiation dose assessment, workflow efficiency, or downstream outcome modeling.

##### Models’ validation, and evaluation

Validation of imaging AI models was variable, primarily relying on comparison with expert manual contours in 22/34 (65%) of studies. Additional external or independent validation dataset approaches (12/34, 35%), physician-based clinical acceptability assessment (5/34, 15%), outcome-based evaluation (21%), and dosimetric validation (29%) were also applied.

Performance assessment was mainly reported by calculating the Dice Similarity Coefficient (DSC) to evaluate the accuracy of AI-driven auto-segmentation of structures in approximately 55 to 60% of studies. Overall median DSC across cardiac structures was approximately 0.85 (range of 0.76–0.94). Performance was strong with large-structures auto-segmentation, with whole-heart (median DSC: 0.93–0.95), cardiac chambers (median DSC: 0.88–0.92) and great vessels (Median DSC: 0.80–0.88) segmentation demonstrating the highest agreement. In contrast, smaller structures such as coronary arteries showed lower performance and greater variability. Coronary artery segmentation was a clear outlier, with lower DSC values (0.56–0.65) and occasional near-zero overlap for distal segments, reflecting the technical challenges of segmenting small structures on non-contrast CT. One feasibility study Thor et al. [41] intentionally reported very poor performance (DSC 0.15) to highlight current methodological limitations for left anterior descending (LAD) artery segmentation on non-contrast CT.

Among studies that did not report DSC (12/34, 35%), particularly those focused on calcification assessment, alternative validation metrics were used. These included intraclass correlation coefficients (ICC 0.85–0.99) for calcium burden quantification, Cohen’s kappa (κ = 0.85–0.91) for risk stratification, surface distance measures for coronary localization, and dosimetric or outcome-based assessments.

##### Quality of studies

**Fig. 5:**
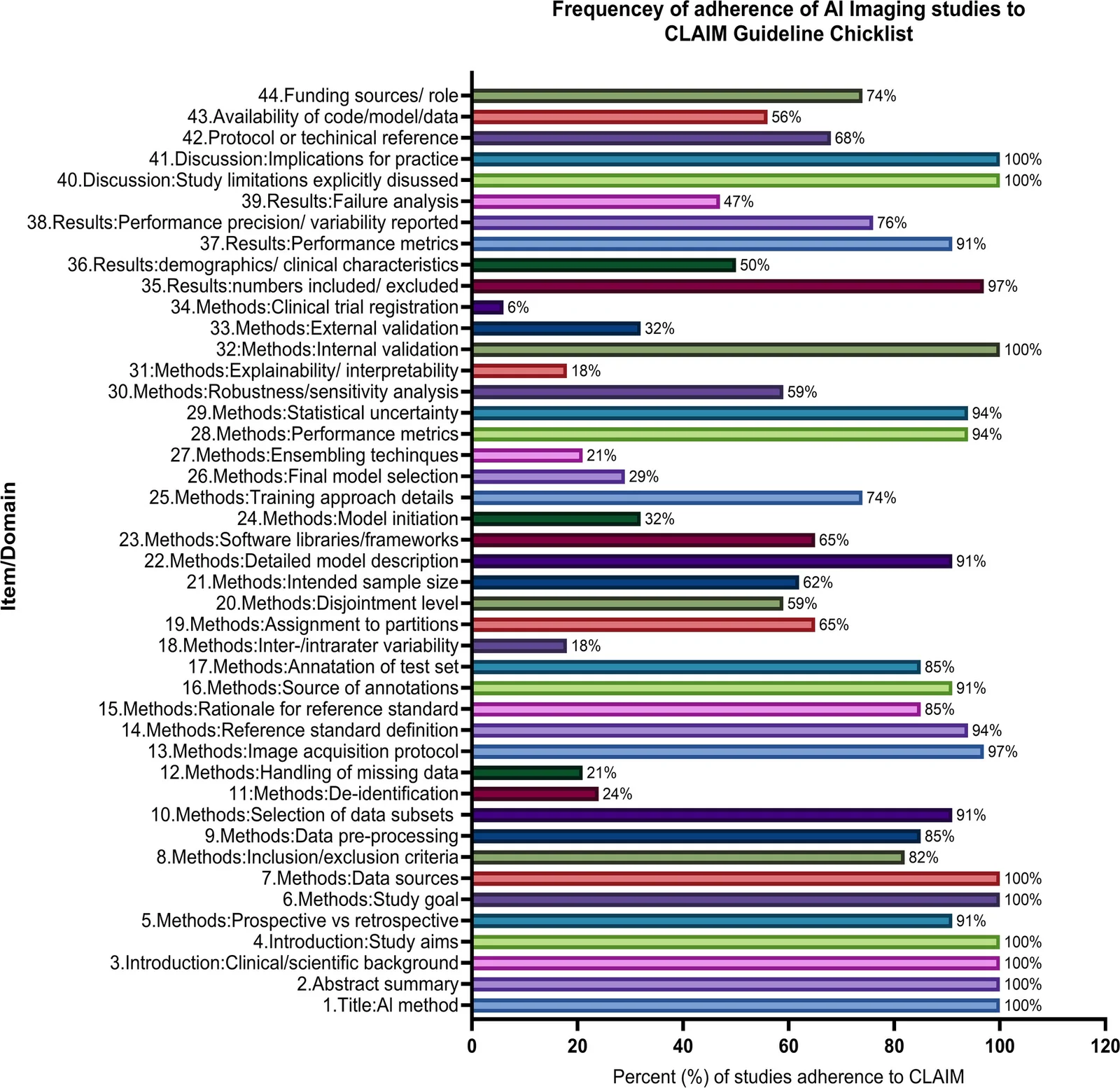
Frequency of adherence of studies applied AI or ML for cardiovascular imaging analysis for patients with cancer receiving RT, to the updated Checklist for Artificial Intelligence in Medical Imaging (CLAIM)

Across the 34 imaging studies, CLAIM adherence averaged 71% across all domains (71%; SD = 29; 95% CI = 62.10–79.90) with 12/34 (35%) falling below 70%. Core methodological components such as title, abstract, clinical background, study aims, data sources, image acquisition protocols, model performance evaluation/metrics, and internal validation procedures demonstrated high reporting compliance (90%-100%). Important methodological and AI-specific elements were inconsistently reported including handling of missing data (7/34, 21%), data de-identification (8/34,24%), inter- or intra-rater variability (6/34, 18%), dataset partitioning details, model transparency, and explainability approaches (6/34,18%). External validation was performed in only 11/34 (32%) studies, and a very limited number of studies reported clinical trials registration (2/34, 8%). Only 56% of studies reported funding sources, while availability of code, models, or data was reported in 74% and 56% of studies, respectively (Fig. 5).

##### Risk-of-bias assessment

**Fig. 6:**
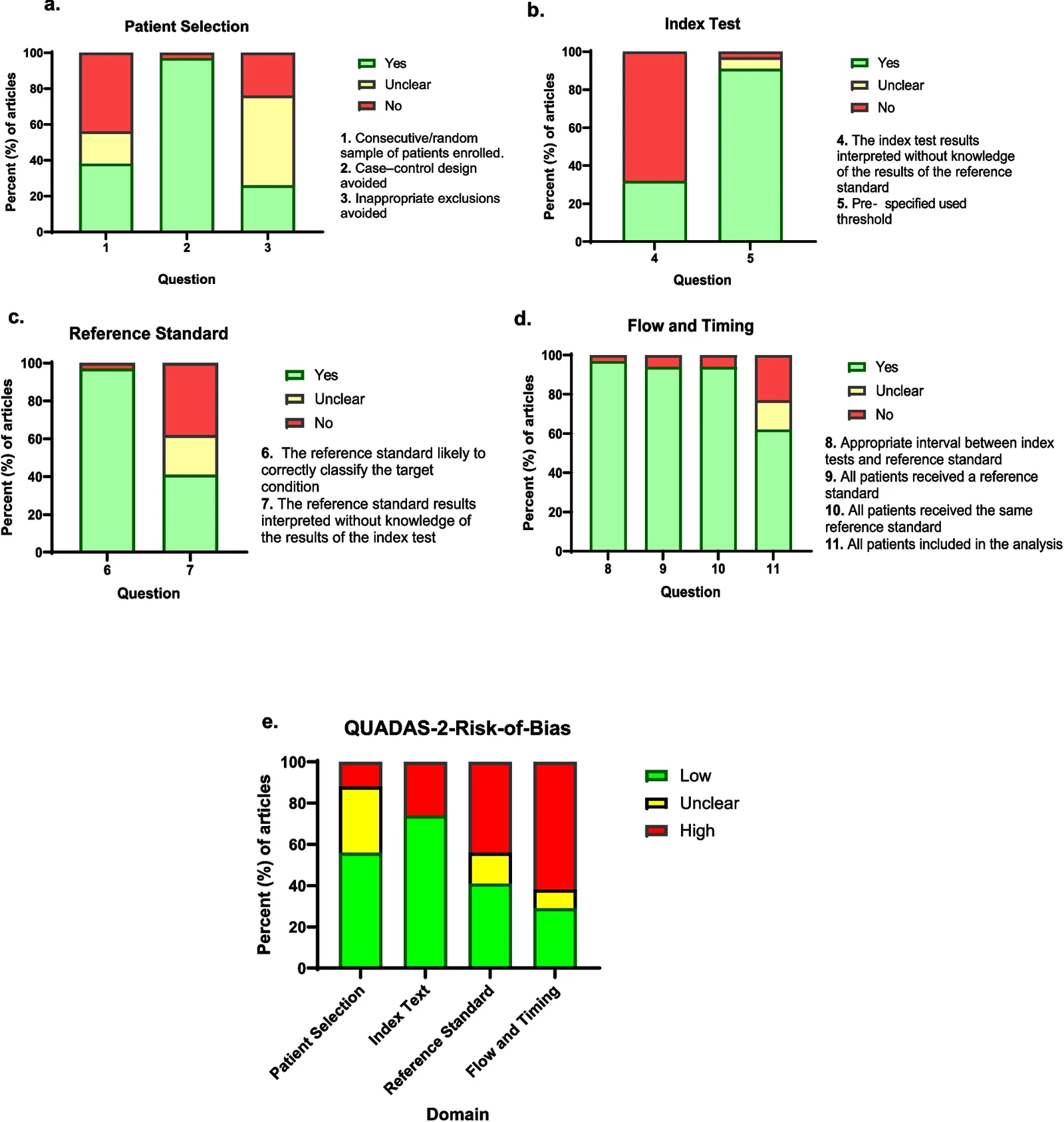
Frequency of Quality Assessment of Diagnostic Accuracy Studies (QUADAS-2) tool across eleven questions covering the four domains: patient selection, index test, reference standard, and flow and timing, across the 34 included AI-based cardiovascular imaging studies. **a** Frequency of answers/assessment of the three questions of the first domain; Patient Selection. **b** Frequency of answers/assessment of the two questions of the second domain; Index Text. **c** Frequency of answers/assessment of the two questions of the third domain; Reference Standard. **d** Frequency of answers/assessment of the four questions of the fourth domain; Flow and Timing. **e** Frequency of overall risk-of-bias of the four domains of QUADAS-2 assessment tool

The overall QUADAS-2 risk-of-bias was high, with 28/34 (82%) studies assessed as high risk-of-bias. Assessment of the four domains across all studies demonstrated that the flow and timing domain demonstrated the lowest overall risk-of-bias, with 21/34 (62%) studies rated as low risk compared with 10/34 (29%) studies rated high risk, and 3/34 (9%) studies rated unclear risk (Fig. 6e). Most studies reported appropriate intervals between index tests and reference standards (97%), and ensured all patients received the same reference standard (94%), although only 62% included all patients in the final analysis (Fig. 6d). In contrast, patient selection showed only 4/34 (12%) of studies at low risk, 11/34 (32%) at unclear risk and 19/34 (56%) at high risk [13/38 (56%) of studies enrolled consecutive or random patient samples and 9/34 (26%) avoided inappropriate exclusions, while 33/34 (97%) avoided case–control designs] (Fig. 6a). For the index test domain, 25/34 (74%) were at high risk [only 11/34 (41%) of studies reported blinded interpretation of model outputs/results relative to the reference standard, although prespecified decision thresholds were reported in 31/34 (91%)] (Fig. 6c). In the reference standard domain, 14/34 (41%) of studies were judged at high risk [most studies employed reference standards likely to correctly classify the target condition (33/34, 97%), however, only 14/34 (41%) reported blinded interpretation of reference annotations relative to AI predictions] (Fig. 6d). Detailed Risk-of-Bias per study was included in Supplementary File S3.

## Discussion

This systematic review of 65 studies mapped the current landscape of AI in RT-associated CVT. AI models showed early potential for identifying patients at high risk of radiation-induced CVT and for optimizing treatment planning to reduce cardiac exposure. However, consistent methodological weakness and heterogeneity limit confidence in these findings. Outcome definitions, validation strategies, and performance reporting varied widely across studies. Calibration was rarely assessed, external validation of predictive values was limited, and adherence to AI reporting standards was incomplete. These methodological gaps may limit the reliability in guiding clinical decisions.

Two main complementary AI application streams emerged: predictive risk modeling and cardiovascular imaging automation. Predictive models targeted clinically meaningful outcomes including MACEs, heart failure, arrhythmias, and composite cardiotoxicity, incorporating clinical, dosimetric, and radiomic variables in ways that go beyond standard dose-volume metrics. Overall, these models demonstrated good discriminatory performance (median AUC of about 0.80), particularly neural network-based approaches, suggesting that AI can capture complex nonlinear interactions between radiation dose distributions and patient-specific risk factors. Discrimination metrics such as AUC reflect a model’s ability to distinguish between patients who will and will not develop cardiovascular toxicity (i.e., event, or no-event) [42]. The median AUC values observed across studies suggest that many of these models achieve clinically meaningful risk stratification between higher- and lower risk patients following RT. Exploratory pooled analyses of selected predictive studies demonstrated an overall accuracy of approximately 0.83 using random-effects modeling, supporting favorable model performance across included studies. Considering substantial heterogeneity in studies, these findings should be interpreted cautiously.

However, discrimination alone does not establish clinical utility. Calibration is essential for determining whether predicted probabilities accurately reflect observed event rates [43]. A model with good AUC can still generate poorly calibrated risk estimates, limiting its usefulness for clinical decisions such as initiating cardioprotective therapy or adjusting surveillance intervals. Interestingly, random forest models tended to demonstrate higher specificity than neural networks, suggesting fewer false-positive predictions. This highlights a potential trade-off in model selection between maximizing detection of cardiovascular toxicity and minimizing unnecessary downstream testing or interventions. A model optimized for sensitivity will flag more patients for cardiac monitoring, potentially burdening low risk patients, while one optimized for specificity may miss patients who would benefit from early intervention. Without prospective outcome data, the right balance cannot be determined for any given clinical setting.

AI imaging studies focused primarily on automated cardiac substructure segmentation and coronary calcification scoring, aiming to improve cardiac dose estimation. Deep learning models showed strong performance for larger structures: median DSC 0.93–0.95 for the whole heart and 0.88–0.92 for cardiac chambers. This level of accuracy can reduce interobserver variability and support more consistent dosimetric assessment in treatment planning [44]. Arjmandi et al., demonstrated that DL-automated contouring approach reduced the inter-expert variability and increased dosimetric accuracy in prostate cancer RT planning, compared to the gold standard reference contouring [44]. Better cardiac delineation is particularly relevant for thoracic malignancies such as those of the breast, lung, and esophageal cancers, where heart-sparing strategies depend on precise structure contouring. However, segmentation accuracy drops for small, low-contrast structures such as the coronary arteries [45, 46]. This limitation is clinically significant because ischemic heart diseases caused by coronary artery disease are a common mechanism of radiation-induced cardiac morbidity [47, 48]. Higher total RT doses, larger irradiated cardiac volumes, and preexisting cardiovascular comorbidities were identified as major risk factors of coronary artery disease after RT [10, 47, 49]. Darby et al. showed that even modest increases in mean heart dose increase ischemic heart disease rates in breast cancer patients, underscoring the importance of coronary-level precision [47].

Non-contrast CT, motion artifacts, small vessel caliber, and limited annotated datasets continue to hinder reliable coronary segmentation [50]. Current AI imaging tools support global cardiac dose assessment, but coronary-specific risk modeling requires further development. Emerging deep learning architectures including U-Net variants with structural priors, multi-scale feature learning, and noise-robust training strategies show promise for improving small vessel segmentation 51–55. Collectively, this reflects a shift toward structure-aware and noise-resilient modeling that may improve coronary segmentation in clinical CT imaging, though their clinical relevance in RT-associated CVT remains to be classified.

Nearly all predictive studies were rated high risk-of-bias by PROBAST, and most imaging studies were rated high risk by QUADAS-2. These are not marginal quality concerns. In cardio-oncology, where AI predictions could influence lifelong cardiac monitoring or modification of cancer treatment, the analytical quality of a model matters as much as its performance metrics. Limited adherence to TRIPOD + AI and CLAIM guidelines revealed further weaknesses in transparency and reproducibility. Few models addressed class imbalance, demographic diversity, or health equity, raising real questions about generalizability to older, multi-morbid patient populations who make up most of the cardio-oncology clinical population.

Clinically, the field is moving from proof-of-concept research toward translational integration; however, several barriers must be addressed before routine clinical implementation. Most studies were retrospective and single-institutional. Few studies have linked AI outputs to clinical decision pathways or tested models in real workflow settings. For AI to meaningfully change cardio-oncology practice, future work must prioritize external validation, multi-institutional datasets, standardized CVT endpoint definitions, and integration with existing cardiac surveillance frameworks.

## Future directions

Importantly, future collaboration between radiation oncologists, cardiologists, radiologists, imaging scientists, and data scientists will be essential to ensure that AI tools address clinically relevant endpoints and fit within survivorship care models. In addition, future research should prioritize model calibration, external validation in real-world data, and clinical utility analyses to determine whether AI predictions translate into improved patient outcomes. Development of shared, high-quality annotated imaging datasets, particularly for coronary artery structures, will also be critical to advance precision cardiac dose modeling. Finally, embedding AI tools into prospective clinical trials and survivorship programs will be necessary to establish real-world effectiveness and safety before routine clinical adoption.

## Limitations

This review has several limitations. First, heterogeneity existed across included studies in cancer types, cardiovascular endpoints, AI models, and performance metrics, limiting the ability to conduct comprehensive quantitative synthesis. Although exploratory meta-analytic analyses of selected performance measures were performed, interpretation of these pooled estimates should be approached cautiously because variability across studies may influence the reliability and generalizability of summary estimates. Additionally, many studies lacked complete reporting of performance variability measures and diagnostic classification parameters, limiting inclusion of all studies in pooled analyses and increasing the potential for selection bias within exploratory quantitative estimates. Second, many studies lacked complete methodological reporting, making risk-of-bias assessment dependent on available descriptions and potentially underestimating weaknesses. Finally, reported performance metrics may overestimate real-world effectiveness because most studies relied on internal validation and retrospective datasets instead of prospective and external validation studies, which are prone to overfitting and spectrum bias.

## Conclusion

Artificial intelligence applications in radiation-associated cardio-oncology are expanding rapidly and show promising ability to enhance cardiac risk prediction and imaging-based radiation dose optimization. However, current evidence is limited by methodological weaknesses, heterogeneity, insufficient validation, and incomplete reporting. Strengthening study design, transparency, and clinical integration will be critical for transforming AI from a research tool into a reliable component of personalized cardiovascular care for cancer survivors.

## Supplementary Information

Supplementary Material 1.

Supplementary Material 2.

Supplementary Material 3.

Supplementary Material 4. Tables (1-4).

Supplementary Material 5. Figures.

## Data Availability

All data extracted from included studies, demographics and characteristics, data of AI models performance, PROBAST, QUADAS-2 risk-of-bias, CLAIM and TRIPOD + AI are included in Supplementary File S3. Original articles extracted from Rayyan AI tool are available from the corresponding author.

## Abbreviations

3D: Three-dimensional
ACE: Acute coronary event
AF: Atrial fibrillation
AI: Artificial intelligence
AUC: Area under the curve (ROC-AUC)
CI: Confidence interval
CLAIM: Checklist for Artificial Intelligence in Medical Imaging
CNN: Convolutional Neural Network
CT: Computed Tomography
CCTA: Coronary computed tomography angiography
CTCAE: Common Terminology Criteria for Adverse Events
CVT: Cardiovascular toxicity
DL: Deep Learning
DSC: Dice similarity coefficient
EMBASE: Excerpta Medica database
Grad-CAM: Gradient-weighted Class Activation Mapping
H–L: Hosmer–Lemeshow (goodness-of-fit test)
ICC: Intraclass correlation coefficient
ID: Identifier
IQR: Interquartile range
k: Number of folds (k-fold cross-validation)
LAD: Left anterior descending (coronary artery)
MACE: Major adverse cardiac event(s)
MEDLINE: Medical Literature Analysis and Retrieval System Online
ML: Machine Learning
NSCLC: Non-small cell lung cancer
NTCP: Normal tissue complication probability
OS: Overall survival
PCI: Percutaneous coronary intervention
PRISMA: Preferred Reporting Items for Systematic Reviews and Meta-Analyses
PROBAST: Prediction Model Risk of Bias Assessment Tool
PROBAST-AI: PROBAST extension for artificial intelligence
PROSPERO: International Prospective Register of Systematic Reviews
QoL: Quality of life
QUADAS-2: Quality Assessment of Diagnostic Accuracy Studies, version 2
QUADAS-AI: QUADAS extension for artificial intelligence
ROC: Receiver operating characteristic
ROC-AUC: Area under the receiver operating characteristic curve
RT: Radiation therapy
SD: Standard deviation
SHAP: Shapley Additive Explanations
TRIPOD: Transparent Reporting of a multivariable prediction model for Individual Prognosis or Diagnosis
TRIPOD-AI: TRIPOD extension for artificial intelligence
TRIPOD + AI: TRIPOD + AI reporting guideline
U-Net: U-shaped convolutional neural network architecture
