# Supplementary material for: A systematic review of artificial intelligence in radiotherapy associated cardiovascular toxicity
Source: Cardiooncology. 2026 Jun 19;12:110. doi: 10.1186/s40959-026-00528-5 (PMC13543590; doi:10.1186/s40959-026-00528-5)
Supplement: Supplementary file 1 [file 40959_2026_528_MOESM1_ESM.pdf]

PubMedSearchHistory (2)

Search number	Query	Sort By	Filters
8	#1 AND #2 AND #3		Free full text, Full text, English, Humans
7	#1 AND #2 AND #3		Free full text, Full text, English
6	#1 AND #2 AND #3		Free full text, Full text
5	#1 AND #2 AND #3		Free full text
4	#1 AND #2 AND #3		
3	Cardiotoxicity OR Cardiovascular Diseases OR Heart Diseases OR Radiation Injuries OR cardiovascular toxicity OR cardiac toxicity OR heart disease OR radiation-induced heart disease OR RIHD OR radiation-induced cardiovascular disease OR cardiovascular imaging OR Ca		
2	Radiotherapy OR Radiotherapy, Adjuvant OR Radiotherapy, Intensity-Modulated OR radiation therapy OR radiation oncology OR radiochemotherapy		
1	Artificial Intelligence OR Machine Learning OR Deep Learning OR Neural Networks OR AI		

Search Details	Results	Time	Date
(("artificial intelligence"[MeSH Terms] OR ("artificial"[All Fields] AND "intelligence"[All Fields]) OR "artificial intelligence"[All Fields] OR ("machine learning"[MeSH Terms] OR ("machine"[All Fields] AND "learning"[All Fields]) OR "	856	19:11:42	2025/10/01
(("artificial intelligence"[MeSH Terms] OR ("artificial"[All Fields] AND "intelligence"[All Fields]) OR "artificial intelligence"[All Fields] OR ("machine learning"[MeSH Terms] OR ("machine"[All Fields] AND "learning"[All Fields]) OR "	1,375	19:11:32	2025/10/01
(("artificial intelligence"[MeSH Terms] OR ("artificial"[All Fields] AND "intelligence"[All Fields]) OR "artificial intelligence"[All Fields] OR ("machine learning"[MeSH Terms] OR ("machine"[All Fields] AND "learning"[All Fields]) OR "	1,412	19:11:23	2025/10/01
(("artificial intelligence"[MeSH Terms] OR ("artificial"[All Fields] AND "intelligence"[All Fields]) OR "artificial intelligence"[All Fields] OR ("machine learning"[MeSH Terms] OR ("machine"[All Fields] AND "learning"[All Fields]) OR "	1,412	19:11:16	2025/10/01
("artificial intelligence"[MeSH Terms] OR ("artificial"[All Fields] AND "intelligence"[All Fields]) OR "artificial intelligence"[All Fields] OR ("machine learning"[MeSH Terms] OR ("machine"[All Fields] AND "learning"[All Fields]) OR "r	2,299	19:11:06	2025/10/01
"cardiotoxic"[All Fields] OR "cardiotoxicity"[MeSH Terms] OR "cardiotoxicity"[All Fields] OR "cardiotoxicities"[All Fields] OR "cardiotoxity"[All Fields] OR ("cardiovascular diseases"[MeSH Terms] OR ("cardiovascular"[All Fields	3,391,340	19:10:49	2025/10/01
"radiotherapy"[MeSH Terms] OR "radiotherapy"[All Fields] OR "radiotherapies"[All Fields] OR "radiotherapy"[MeSH Subheading] OR "radiotherapy s"[All Fields] OR ("radiotherapy, adjuvant"[MeSH Terms] OR ("radiotherapy"[A	654,826	19:10:37	2025/10/01
"artificial intelligence"[MeSH Terms] OR ("artificial"[All Fields] AND "intelligence"[All Fields]) OR "artificial intelligence"[All Fields] OR ("machine learning"[MeSH Terms] OR ("machine"[All Fields] AND "learning"[All Fields]) OR "n	1,650,413	19:10:25	2025/10/01

Search Name: AI Cardio Toxicity

Date Run: 10/01/2025 01:21:50

Comment:

ID	Search	Hits
#1	Artificial Intelligence OR Machine Learning OR Deep Learning OR Neural Networks OR AI	20819
#2	Radiotherapy OR Radiotherapy, Adjuvant OR Radiotherapy, Intensity-Modulated OR radiation therapy OR radiation oncology OR radiochemotherapy	57533
#3	Cardiotoxicity OR Cardiovascular Diseases OR Heart Diseases OR Radiation Injuries OR cardiovascular toxicity OR cardiac toxicity OR heart disease OR radiation-induced heart disease OR RIHD OR radiation-induced cardiovascular disease OR cardiovascular imaging OR Cardio-oncology	121455
#4	#1 AND #2 AND #3	95

Embase

Session Results

....

No.	Query Results	Results	Date
#5.	#4 AND 'human'/de	240	1 Oct 2025
#4.	#1 AND #2 AND #3	253	1 Oct 2025
#3.	((((((((cardiotoxicity OR cardiovascular) AND diseases OR heart) AND diseases OR radiation) AND injuries OR cardiovascular) AND toxicity OR cardiac) AND toxicity OR heart) AND disease OR 'radiation induced') AND heart AND disease OR rihd OR 'radiation induced') AND cardiovascular AND disease OR cardiovascular) AND imaging OR 'cardio oncology'	252,799	1 Oct 2025
#2.	((((radiotherapy OR radiotherapy,) AND adjuvant OR radiotherapy,) AND 'intensity modulated' OR radiation) AND therapy OR radiation) AND oncology OR radiochemotherapy	454,514	1 Oct 2025
#1.	'artificial intelligence'/exp OR 'artificial intelligence' OR (artificial AND ('intelligence'/exp OR intelligence)) OR 'machine learning'/exp OR 'machine learning' OR (('machine'/exp OR machine) AND ('learning'/exp OR learning)) OR 'deep learning'/exp OR 'deep learning' OR (deep AND ('learning'/exp OR learning)) OR 'neural networks'/exp OR 'neural	983,525	1 Oct 2025

networks' OR (neural AND networks) OR ai

....

history

Recent queries in mesh			
Search	Query	Items found	Time
#4	Search ((#1) AND #2) AND #3	0	19:30:34
#3	Search (Cardiotoxicity OR Cardiovascular Diseases OR Heart Diseases OR Radiation Injuries OR cardiovascular toxicity OR cardiac toxicity OR heart disease OR radiation-induced heart disease OR RIHD OR radiation-induce	32	19:30:16
#2	Search (Radiotherapy OR Radiotherapy, Adjuvant OR Radiotherapy, Intensity-Modulated OR radiation therapy OR radiation oncology OR radiochemotherapy)	22	19:30:03
#1	Search (Artificial Intelligence OR Machine Learning OR Deep Learning OR Neural Networks OR AI)	66	19:29:55

Web of Science Search Strategy (v0.1)

Database: Web of Science Core Collection

Entitlements:

- WOS.SCI: 1992 to 2025
- WOS.AHCI: 2008 to 2025
- WOS.ESCI: 2020 to 2025
- WOS.SSCI: 2008 to 2025

Searches:

1: ALL=(Artificial Intelligence OR Machine Learning OR Deep Learning OR Neural Networks OR AI) Date Run: Wed Oct 01 2025 18:05:12 GMT-0500 (Central Daylight Time) Results: 1819341

2: ALL=(Radiotherapy OR Radiotherapy, Adjuvant OR Radiotherapy, Intensity-Modulated OR radiation therapy OR radiation oncology OR radiochemotherapy) Date Run: Wed Oct 01 2025 18:05:26 GMT-0500 (Central Daylight Time) Results: 533687

3: ALL=(Cardiotoxicity OR Cardiovascular Diseases OR Heart Diseases OR Radiation Injuries OR cardiovascular toxicity OR cardiac toxicity OR heart disease OR radiation-induced heart disease OR RIHD OR radiation-induced cardiovascular disease OR cardiovascular imaging OR Cardio-oncology) Date Run: Wed Oct 01 2025 18:05:45 GMT-0500 (Central Daylight Time) Results: 1222581

4: #1 AND #2 AND #3 Date Run: Wed Oct 01 2025 18:06:02 GMT-0500 (Central Daylight Time) Results: 499

5: #1 AND #2 AND #3 and Article (Document Types) and English (Languages) Date Run: Wed Oct 01 2025 18:06:23 GMT-0500 (Central Daylight Time) Results: 391

6: #1 AND #2 AND #3 and Article (Document Types) and English (Languages) and Article (Document Types) Date Run: Wed Oct 01 2025 18:06:28 GMT-0500 (Central Daylight Time) Results: 391
