# Supplementary material for: A systematic review of artificial intelligence in radiotherapy associated cardiovascular toxicity
Source: Cardiooncology. 2026 Jun 19;12:110. doi: 10.1186/s40959-026-00528-5 (PMC13543590; doi:10.1186/s40959-026-00528-5)
Supplement: Supplementary file 3 [file 40959_2026_528_MOESM5_ESM.pdf]

Supplementary Figure 1

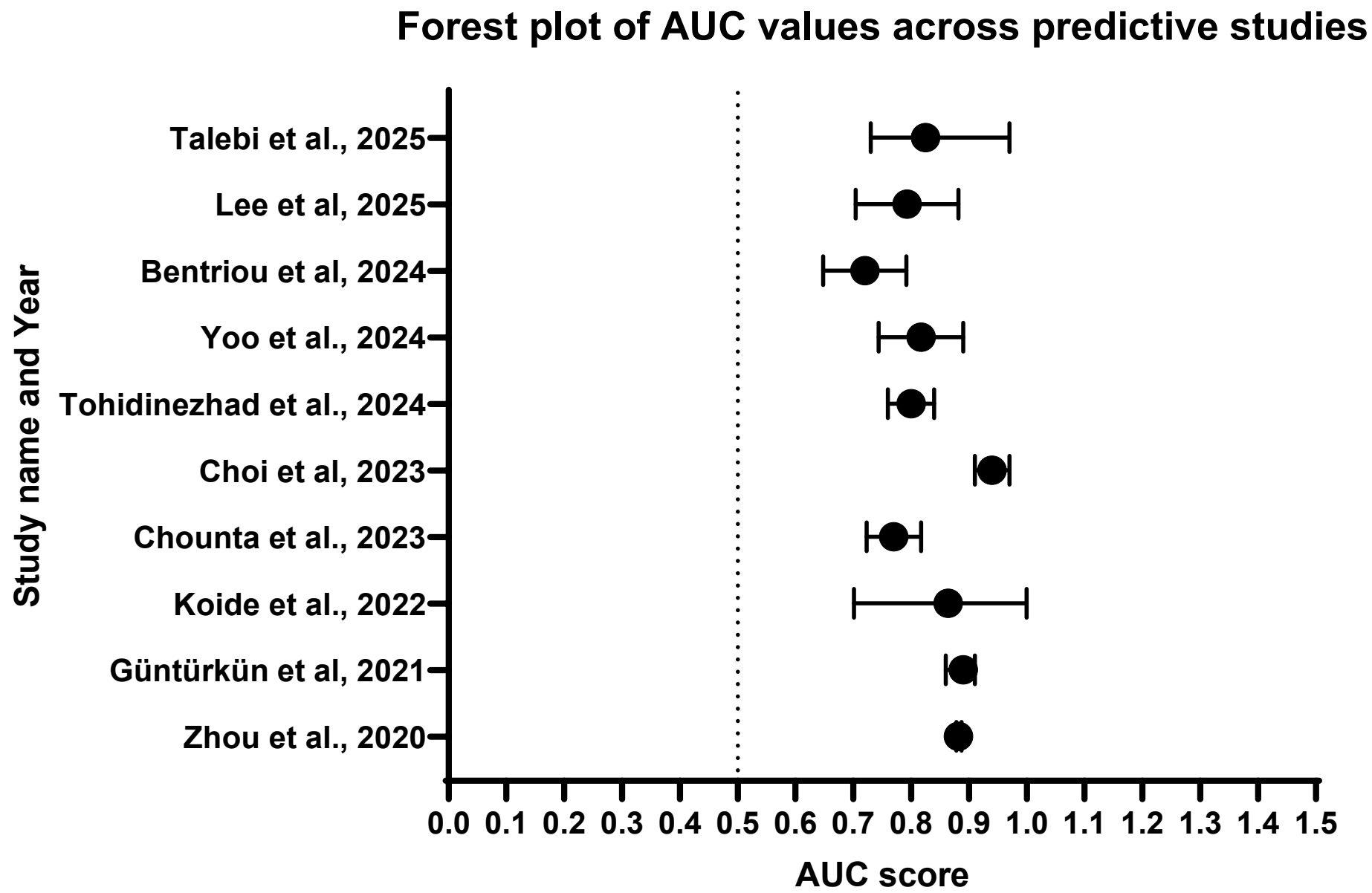

Supplementary Figure 1: Forest-style plot of reported AUC values for AI predictive models used in radiotherapy-associated cardiovascular toxicity studies. Individual studies are presented by first author and publication year on the Y-axis. Squares indicate reported highest AUC estimates and horizontal lines represent corresponding 95% confidence intervals. The vertical dashed line at AUC = 0.50 indicates no discriminatory ability (equivalent to random prediction). Higher AUC values indicate better model performance.

Supplementary Figure 2

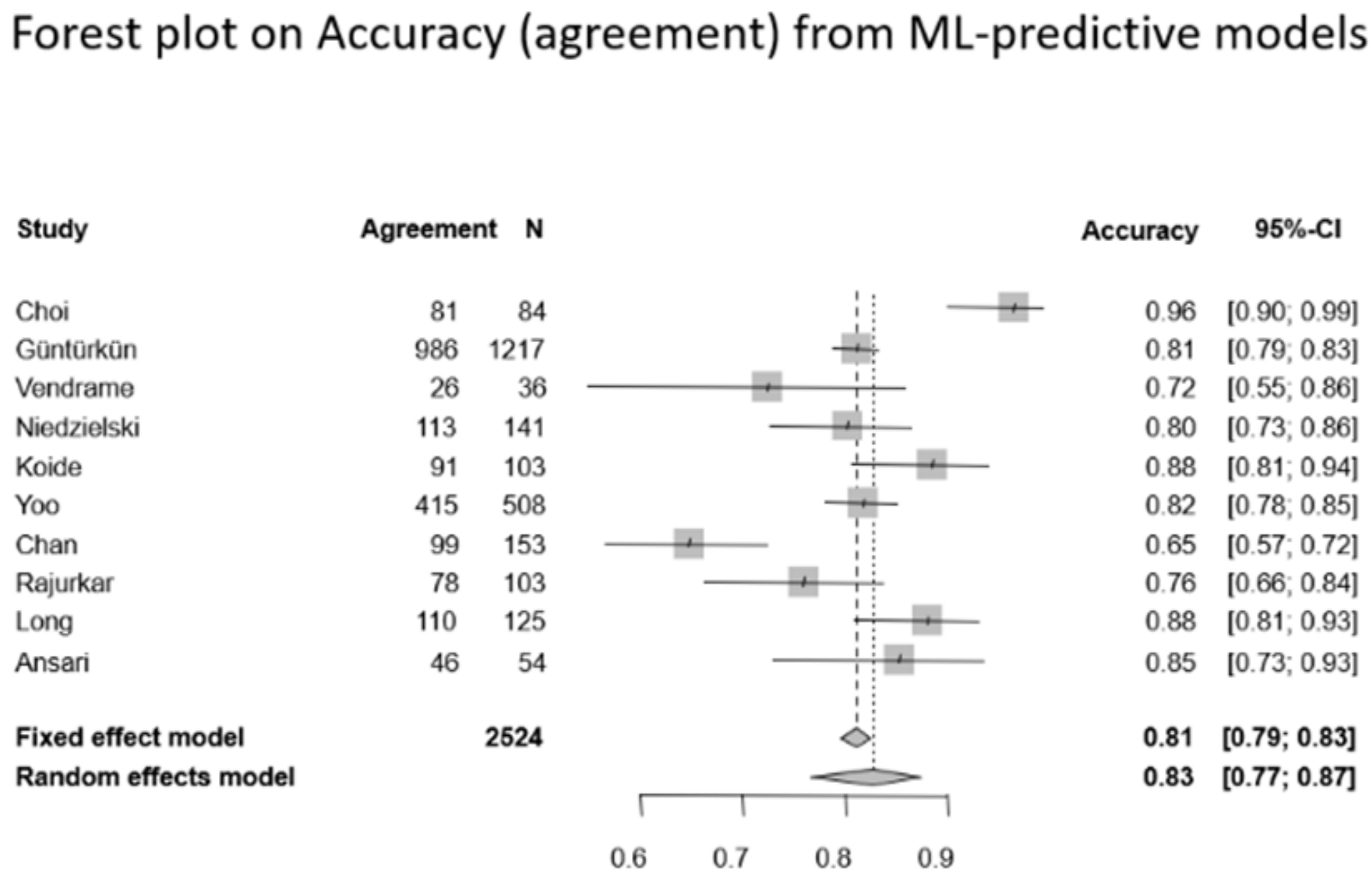

Supplementary Figure 2: Forest plot summarizing the reported classification accuracy of machine learning predictive models for radiotherapy-associated cardiovascular toxicity. Individual studies are represented by squares, with square size proportional to study weight, and horizontal lines representing corresponding 95% confidence intervals. Diamonds indicate pooled estimates using fixed-effects and random-effects models. The pooled random-effects estimate demonstrated an overall classification accuracy of 0.83 (95% CI: 0.77–0.87), indicating generally good predictive performance across included studies despite variability among individual studies.
