# Supplementary material for: A systematic review of artificial intelligence in radiotherapy associated cardiovascular toxicity
Source: Cardiooncology. 2026 Jun 19;12:110. doi: 10.1186/s40959-026-00528-5 (PMC13543590; doi:10.1186/s40959-026-00528-5)
Supplement: Supplementary file 4 [file 40959_2026_528_MOESM4_ESM.zip › Supplementary Table 1.docx]

| Supplementary Table 1: Demographics and characteristics of all included studies applied AI and ML in cardiovascular imaging and cardiovascular toxicity after RT. | | | | | | | | |
| --- | --- | --- | --- | --- | --- | --- | --- | --- |
| **Author, Year** | **Journal** | **Type of cancer** | **Number of participants** | **AI Type** | **Variables** | **Outcome/**  **output** | **AI application** | **Results/**  **conclusion** |
| Wu et al., 2018 ^(1)^ | Medical image analysis | Unspecific | 19 | Generative model: Deep Boltzmann Machine | Cine MRI cardiac contours | Heart segmentation + motional tracking | Imaging/heart motion | AI-driven segmentation accurately tracks heart motion on cine MRI and outperforms existing methods; useful for RT cardiac motion assessment |
| Gernaat et al., 2018 ^(2)^ | Radiotherapy and Oncology | Breast cancer | 2289 | Deep Learning (convolutional neural network (CNN)) | RT planning computed tomographic (CT) scans | Quantified calcifications in coronary arteries (CAC) and thoracic aorta (TAC) | Imaging/Calcification | Deep learning is a reliable method to automatically measure CAC and TAC on RT breast CT scans. |
| Emaus et al., 2019 ^(3)^ | BMJ Open | Breast cancer | 16,000 | Deep Learning | Automatically measured coronary, aortic, and valvular calcifications on RT planning CT; traditional CVD risk factors; treatment characteristics | Incident fatal and non-fatal CVD events | Imaging/Calcification | Automated cardiovascular calcification measurements on routine RT planning CT scans can improve CVD risk prediction in breast cancer patients before potentially cardiotoxic therapy. |
| Morris et al., 2020 | Medical Physics | Breast cancer | 32 | Deep Learning | Cardiac substructure volumes (12 substructures: chambers, great vessels, coronary arteries, pulmonary veins), consensus clinical scores | Accuracy of cardiac substructure segmentation; clinical acceptance | Imaging/Segmentation | Deep learning enables rapid, accurate segmentation of cardiac substructures, improving efficiency and potentially facilitating cardiac-sparing radiotherapy. |
| Van Velzen et al., 2020 ^(4)^ | Radiology | Unspecific | 7240 | Deep Learning (convolutional neural network (CNN)) | Non-contrast CT images (CAC scoring CT, chest CT, PET attenuation CT, RT planning CT, low-dose chest CT), manual Agatston CAC and TAC scores | Coronary artery calcium (CAC) and thoracic aorta calcium (TAC) quantification; cardiovascular disease risk category | Imaging/Calcification | DL calcium scoring is robust across multiple CT protocols; enabling reliable CVD risk stratification from diverse CT exams |
| Niedzielski et al., 2020 ^(5)^ | Acta Oncologica | Lung cancer | 141 | Regression model | Dose-volume histogram (DVH) metrics for whole heart and substructures (mean, max dose, V5Gy–V70Gy); pericardial effusion grade ≥2 (CTCAE v4.0) | Radiation-induced pericardial effusion (PCE) | Prediction/Dosiomics | Including cardiac substructure dose metrics improve prediction of radiation-induced pericardial effusion in NSCLC patients; elastic net regression is effective for this toxicity modeling |
| Chan et al., 2020 ^(6)^ | Clinical Lung Cancer | Lung cancer | 153 | Neural Network | Cardiac radiation doses to 15 cardiac substructures and whole heart; 74 input features in ANN | Overall survival related to radiation induced cardiac toxicity | Prediction/Dosiomics | Higher radiation doses to cardiac substructures are associated with decreased OS in early-stage NSCLC treated with SABR |
| Loap et al., 2020 ^(7)^ | Cancer Radiothérapie | Breast cancer | 40 | Deep Learning | CT Scans for Cardiac substructure contours | Cardiac substructure contours (whole heart, chambers, left ventricle walls) | Imaging/Segmentation | Cardiac AI-based autosegmentation showed acceptable performance for cardiac cavity delineation |
| Morris et al., 2020 ^(8)^ | Journal of Applied Clinical Medical Physics | Thoracic Cancers | 16 | Deep Learning (U-Net) | CT-RT planning (CT-SIM) and MRI (MR-linac) | 13 cardiac substructures; mean and maximum doses; LV-V5; plan complexity (MU, treatment time) | Imaging/Segmentation | Integration of MRI with deep learning contouring allows improved cardiac substructure sparing in RT planning potential to reduce radiation-related cardiotoxicity |
| Zhou et al., 2020 ^(9)^ | Journal of the American Heart Association | Multiple cancers | 4309 | Classification models: (k-nearest neighbors, LR, SVM, RF, and GB) | Clinical variables (age, hypertension, glucose levels, left ventricular ejection fraction, creatinine, aspartate aminotransferase), laboratory tests, echocardiographic measurements | Cancer therapy–related cardiac dysfunction (CTRCD): 6 cardiovascular outcomes: coronary artery disease, atrial fibrillation, heart failure, stroke, myocardial infarction, and de novo CTRCD | Prediction | ML can stratify cardiac risk in oncology patients using longitudinal clinical and lab data, enabling early detection and prevention of therapy-related cardiac dysfunction |
| Xu et al., 2020 ^(10)^ | Journal of Applied Clinical Medical Physics | Breast cancer | 40 | Knowledge-based planning (KPB) using kernel density estimation (KDE) DVH prediction models | Geometric relationship features + prior high-quality IMRT plans; breathing condition (Free breathing vs abdominal DIBH), structures include PTV and OARs (heart, LADCA, left lung). | DVHs for OARs and corresponding auto-optimized KDE plans generated from those DVHs | Prediction/Dosiomics | KDE-based KBP can predict DVHs and generate clinically comparable plans when trained/applied within the same breathing condition, but performance degrades across breathing conditions |
| Güntürkün et al, 2021 ^(11)^ | JCO Clinical Cancer Informatics | Childhood Cancer | 1217 | Machine Learning and Deep Learning (Extreme Gradient Boosting [XGBoost], Convolutional Neural Networks [CNN], and Genetic Algorithms for feature selection) | Clinical data and ECG | Cardiomyopathy after RT | Prediction | AI using ECG data can assist in the early identification of childhood cancer survivors at increased risk for future cardiomyopathy, potentially enabling earlier intervention |
| Harms et al., 2021 ^(12)^ | Medical Physics | Lung cancer | 55 | Deep Learning (convolutional neural network (CNN)) | Computed Tomography (CT) scans (33 with contrast, 22 without contrast) | Automatic generation of contours for the whole heart and 15 cardiac substructures | Imaging/Segmentation | The deep learning network successfully automates the delineation of cardiac substructures from CT alone, providing a tool to better understand and potentially mitigate radiation-induced cardiac toxicities |
| Garrett Fernandes et al., 2021 ^(13)^ | Radiotherapy and Oncology | Lung cancer | 177 | Neural network | Planning CT images (contrast and non-contrast), expert contours of heart, cardiac chambers, and great vessels | Geometric contour accuracy, dosimetric parameter estimation, clinical acceptability | Imaging/Segmentation | The AI model provides accurate, consistent, and time-efficient contours of cardiac substructures on RT planning CTs |
| Zeleznik et al., 2021 ^(14)^ | NPJ Digital Medicine | Breast cancer | 6535 | Deep Learning | RT-planning CT scans | Automatic volumetric heart segmentation time, concordance, failure rate (<0.85 Dice) | Imaging/Segmentation | Deep-learning volumetric heart segmentation improves efficiency, consistency, and reliability for RT planning in breast cancer |
| Gal et al., 2021 ^(15)^ | JAMA Oncology | Breast cancer | 15915 | Deep Learning | CAC scores from planning CT scans (Agatston risk categories: 0, 1-10, 11-100, 101-399, >400), treatment factors, age, calendar year | Cardiovascular disease (CVD) and coronary artery disease (CAD) incidence | Imaging/Calcification | Automated CAC quantification on radiotherapy planning CT is associated with increased CVD/CAD risk in breast cancer patients and can help identify high-risk individuals for preventive strategies |
| van Velzen et al., 2022 ^(16)^ | International Journal of Radiation Oncology Biology Physics | Breast cancer | 31 | Convolutional neural networks (CNNs) | RT planning CTs | 1. segmentation of the cardiac chambers and large arteries, 2. Localization of coronary arteries. 3. Dosimetric parameters analysis | Imaging/Dosiomics | CNN can automatically obtain accurate estimates of planned radiation dose and  dosimetric parameters for the cardiac chambers, large arteries, and coronary arteries.” |
| Van Velzen et al., 2022 ^(17)^ | International Journal of Radiation Oncology Biology Physics | Breast cancer | 5300 | Deep Learning (CNN) for autosegmentation and Linear regression for prediction. | RT planning computed tomographic (CT) scans | 1. Image autosegmentation of cardiovascular structures and coronary calcium scores (CAC) detection. 2. Dose volume parameters extraction. 3.hospitalization for or death from ischemic HD | Imaging/Dosiomics | Radiation exposure of cardiac structures is associated with increased risk of HD. AI-automatic segmentation of cardiac structures enables spatially localized dose estimation, which can prevent RT−induced cardiac damage. |
| Kim et al., 2022 ^(18)^ | The Breast | Breast cancer | 24000 | Deep learning | RT-planning CT scans | Accuracy of the automated CAC scoring method (T-scores) compared to the gold standard (C-scores) | Imaging/Calcification | The automated deep learning method accurately measures CAC on low-dose breast radiotherapy planning CT scans, for risk stratification and for future individualized treatment planning. |
| Van den Oever et al., 2022 ^(19)^ | Journal of Medical Systems | Breast cancer | 24 | Deep Learning | CT scans | Accuracy and efficiency of automated contouring for 8 cardiac structures and the lungs | Imaging/Segmentation | The deep learning models can automatically contour key cardiac structures with acceptable accuracy and high efficiency, for improving cardiac dose constraint evaluation in RT |
| Koide et al., 2022 ^(20)^ | Scientific Reports | Breast cancer | 103 | Convolutional neural network (CNN). + regression model | Anteroposterior and lateral chest X-rays; RT planning parameters | Predicted cardiac dose exposure (mean heart dose) | Prediction | Deep learning chest X-ray model can predict patients likely to benefit from DIBH-RT for reduced cardiac dose; may assist in individualized treatment planning |
| Bitterman et al., 2022 ^(21)^ | International Journal of Radiation Oncology Biology Physics | Lung cancer | 353 | Classification models: Boosting framework, decision trees and regression learning) | RT plan characteristics, lung dose metrics (V20 Gy, V5 Gy, MLD), mean heart dose (MHD) | Potential reduction in MHD and associated lung dose tradeoffs | Prediction/Dosiomics | ML-guided decision support can identify RT plans that reduce heart dose with clinically acceptable lung dose tradeoffs, supporting optimization of RT planning for NSCLC |
| Atkins et al., 2022 ^(22)^ | JCO Clinical Cancer Informatics | Lung cancer | 428 | Deep Learning | Coronary artery calcium (CAC) volume from RT planning CT; lung cancer and cardiovascular covariates | All-cause mortality; major adverse cardiac events (MACEs) | Imaging/Calcification | Automated deep learning can quantify CAC from routine RT planning CTs; elevated CAC predicts increased mortality; supports potential use for cardiac risk screening before cancer therapy |
| Momin et al., 2022 ^(23)^ | Frontiers in Oncology | Lung cancer | 55 | AI based Knowledge-based planning (KBP) model (RapidPlan: ML and DL KBP)) | Cardiac substructure doses (15 substructures including coronary arteries, valves, great vessels, chambers); PTV coverage; other organ-at-risk doses | Cardiac substructure dose sparing (e.g., V15Gy for LAD artery), overall plan quality | Prediction/Dosiomics | Knowledge-based planning with detailed cardiac substructures (CS-KBP) can significantly improve cardiac dose sparing in NSCLC RT while maintaining treatment quality |
| Radici et al., 2022 ^(24)^ | Life-Basel | Multiple cancers | Not specified | Deep Learning (U-Net) | Planning CT scan Organs-at-risk and target volume delineation; geometric performance; dosimetric differences; contouring time | Contouring Time and Accuracy of auto-segmentation vs manual; time savings; dosimetric impact | Imaging/Segmentation | Deep learning auto-segmentation significantly reduces contouring time, simplifies workflow, reduces interobserver variability, and can improve radiotherapy planning efficiency |
| Monin et al., 2022 ^(25)^ | Physics in Medicine and Biology | Thoracic Cancers | 120 | Deep Learning | CT images, manually contoured cardiac substructures, network-derived feature maps | Automatic segmentation of up to 15 cardiac substructures (chambers, coronary arteries, great vessels, valves) | Imaging/Segmentation | Mutual enhancing DL can accurately segment small and large cardiac substructures, facilitating RT planning and potential reduction of CVT through precise dose mapping |
| Smith et al., 2022 ^(26)^ | Medical Physics | Thoracic Cancers | 933 | Deep learning (U-Net) | CT images; clinician-provided contour edits | Automatically generated OAR contours that improve in accuracy over time with user corrections | Imaging/Segmentation | Interactive ML can produce accurate RT contours while continuously improving from clinician input |
| Chun et al., 2022 ^(27)^ | Radiation Oncology | Breast cancer | 59 | Deep Learning | Non-contrast CT (NCT). | Cardiac substructure contours (heart, LA/LV/RA/RV, LAD, RCA). DVH metrics from plans (Dmean, Dmax, V5/V10/V20/V30/V40) | Imaging/Segmentation | DL-generated synthetic contrast CT from NCT is feasible and shows potential utility for cardiac substructure delineation |
| Walls et al., 2022 ^(28)^ | Physics and Imaging in  Radiation Oncology | Lung cancer | 20 | Deep Learning | 3D-CT scans or 4D-AVE CT scans  of the thorax | Auto-segmented cardiac  substructures (RA, LA,  RV, LV, AO, PA, SVC,  IVC, whole heart,  pericardium) | Imaging/Segmentation | Deep learning-based auto-segmentation can accurately delineate cardiac substructures and predict dosimetry  on 4D-CT scans in lung cancer RT |
| Choi et al., 2023 ^(29)^ | Medical Physics | Breast cancer | 84 | Deep learning for auto-segmentation and logistic regression | Planning CT, dose distribution, auto-segmented cardiac structure (LV, LAD, RCA), extracted imaging features and active regions | Auto-segmentation of cardiac structures. Acute coronary events (ACE) prediction | Imaging/Features; Prediction of RT-related cardiac events using CT + dose + substructure features | 1.Integrating CT, dose data, cardiac substructure features using DL framework can accurately predict ACE, following breast RT; 2. substructure features, particularly LV, are critical for ACE risk modeling, supporting personalized risk stratification in breast RT patients |
| Vendrame et al., 2023 ^(30)^ | Applied Sciences | Breast cancer | 36 | Recurrent neural networks (RNN): Deep Bidirectional Long Short-Term Memory (BLSTM) | Respiratory signals/waveforms acquired during pre-treatment CT (superior-inferior and anterior-posterior directions) | Eligibility for Deep Inspiration Breath-Hold (DIBH) radiotherapy | Patient Selection | BLSTM-RNN can classify patients eligible for DIBH with good accuracy from respiratory signals, offering a promising tool for building automated decision systems in RT |
| Walls et al., 2023 ^(31)^ | Radiotherapy and Oncology | Lung cancer | 291 | Deep learning | Statin therapy (high-intensity vs. low/moderate-intensity) 8and mean heart dose (MHD) | Occurrence of clinical cardiovascular events (CVEs), and cardiovascular structures autosegmentation | Imaging/Segmentation | Statin therapy significantly reduces the risk of radiation-induced cardiotoxicity in NSCLC patients. High-intensity statin therapy further reduces the 2-year CIF of CVEs |
| Haseltine et al., 2023 ^(32)^ | Physics and Imaging in Radiation Oncology | Lung cancer | 239 | Deep learning | RT- CT planning | -Auto-segmentation of pericardium and aorta, Cardiac Calcium Burden (CCB).  -Overall survival | Imaging/Calcification | Pre-treatment cardiac calcium burden, easily quantifiable using DL autosegmentation of cardiovascular structures on RT planning CT, is an independent prognostic factor for overall survival in NSCLC patients treated with RT |
| Chounta et al., 2023 ^(33)^ | Cancers | Childhood Cancer | 7670 | Random forest algorithms | Heart-dose distribution, 93 first order and spatial dosiomics features, mean heart dose (MHD) | Radiation-induced valvular heart disease (VHD) | Prediction/Dosiomics | Dosiomics heart signatures, can enhance prediction of radiation-induced VHD in childhood cancer survivors using ML; may inform personalized follow-up and risk stratification |
| Ladbury et al., 2023 ^(34)^ | International Journal of Radiation Oncology Biology Physics | Lung cancer | 544 | Multiple supervised ML classification models: logistic regression, naïve Bayes, k-nearest neighbors, support vector machine, random forest, and extreme gradient boosting (XGBoost); explainability via SHAP (Shapley Additive Explanation) | Clinical and dosimetric features including mean lung/esophagus dose, lung V20, esophagus V20 | Grade ≥3 pulmonary, cardiac, and esophageal toxicities | Prediction/Dosiomics | ML with explainable AI can empirically validate and discover clinically useful dosimetric thresholds for toxicity in NSCLC radiotherapy |
| Lee et al., 2023 ^(35)^ | International Journal of Radiation Oncology, Biology, Physics | Lung cancer | 428 | Explainable Boosting Machine (EBM); multiblock sparse partial least squares regression; supervised PCA; Boruta; random survival forest; extreme gradient boosting; DeepSurv | Dose-volume histogram features of cardiopulmonary substructures (heart chambers, pericardium, lungs), clinical parameters (chemotherapy), overlap volumes with PTV | Overall survival (OS) prediction; selection of dose-volume constraints for cardiopulmonary substructures | Prediction/Dosiomics | Explainable ML (EBM) can identify cardiac dose features impacting OS and guide dose-volume constraints for cardiopulmonary substructures in NSCLC RT |
| Finnegan et al., 2023 ^(36)^ | Physical and Engineering  Sciences in Medicine | Breast and Lung | 330 | Deep learning | RT-planning CT scans | Fully automated segmentation of (Whole heart (WH)Cardiac chambers, Great vessels, Coronary arteries, Heart valves, Conduction nodes) | Imaging/Segmentation | Hybrid segmentation using deep  learning, whole-heart-guided  multi-atlas mapping, and geometric  modelling can accurately and reliably delineate cardiac substructures on RT planning CT scans |
| Monlezun et al., 2023 ^(37, 38)^ | Journal of Cardiovascular  Development and Disease | Thoracic Cancers | 148755036 | Regression model, Neural Network | patient demographics (age, sex, race, insurance type, income level, and geographic  region), clinical comorbidities identified by ICD-10 codes,  mortality risk score, prior RT status | primary output- in-hospital mortality,  secondary outputs- PCI, post–radiation therapy PCI, complications, and total hospitalization cost | Prediction | Persistent disparities  in post-radiation PCI and AMI  care, with significant clinical,  economic, and ethical implications,  supporting targeted interventions to improve equity and outcomes. |
| Chin et al., 2023 ^(39)^ | Clinical Oncology | Lung cancer | 30 | Deep Learning | Planning CT scans and RT plan dose  Outputs compared against expert manual contours | Automatically generated contours for 18 cardiac structures plus corresponding dose metrics (e.g. mean/max dose; DVH agreement) | Imaging/Segmentation | Novel hybrid automatic segmentation tool reported high accuracy and consistency, in cardiovascular substructure  dose calculations  and for future studies on long-term cardiac toxicity |
| Bernasconi et al., 2024 ^(40)^ | Progress in Artificial Intelligence | Breast cancer | 1440 | Causal network model (Causal AI) | Prognostic factors: age, tumor grade, histology, ki67, molecular subtype, vascular invasion, lymph node involvement, tumor dimension. Treatments: neoadjuvant/adjuvant chemotherapy, radiotherapy, molecular targeted therapy, and hormone therapy. | Cardiotoxicity and cardiovascular diseases (CVDs) | Prediction/Explaining Causation – Designed to disentangle long-term treatment impacts and predict individual susceptibility to cardiotoxicity. | Combining clinical expert knowledge with AI causal discovery algorithms allows for the creation of a valid model to predict cardiotoxicity in young breast cancer survivors, even when data is biased or missing. |
| Borges et al., 2024 ^(41)^ | Journal of Medical Imaging and Radiation Sciences | Breast cancer | 39 | Deep learning (CNN) for autosegmentation and Linear regression for DVH | RT planning computed tomographic (CT) scans | 1. Autosegmentation of cardiovascular structures and organs at risks (OAR) (aorta, heart, RT/LT atrium and ventricles, LT breast, LT Lung and cardiac area). 2. Dose volume parameters calculation compare the relationship between the volume of the cardiac area and the volume of the OARs. | Imaging/Segmentation | AI automatic tools can overcome the technical difficulties in Dose-volume assessment protocols in patients with LT Breast cancer considering the cardiac substructures. |
| Chin et al., 2024 ^(42)^ | Clinical Oncology | Lung cancer | 117 | Deep learning | RT-Planning CT. Physical and EQD2 doses to 18 cardiac substructures, mean heart dose (MHD), maximum doses, patient demographics (age, gender) | Overall survival (OS), pericardial effusion | Imaging/Segmentation/  Dosiomics | SABR for lung cancer can deliver high doses to cardiac substructures; median MHD stratifies survival; dose to ascending aorta and gender also impact OS. |
| Thor et al., 2024 ^(43)^ | Advances in Radiation Oncology | Breast cancer | 65 | Convolutional neural networks (CNNs), and Linear regression for association | RT-planning CT. Mean dose to 9 cardiac substructures, ipsilateral and contralateral lungs, cancer laterality | Autosegmentation of 9 cardiovascular substructures and dose extraction. Association between substructure doses and cardiorespiratory fitness (linear regression) | Imaging/Dosiomics | Modern breast cancer RT minimizes cardiopulmonary doses; laterality and RT technique affect substructure doses, but these doses are not correlated with cardiorespiratory fitness |
| Mohan et al., 2024 ^(44)^ | Indian Journal of Medical and Paediatric Oncology | Breast cancer | 9 | Deep Learning | CT images | Autosegmentation of LAD artery, LAD imaging features, contour size, interpatient variability | Imaging/Segmentation | LAD artery autosegmentation is challenging due to small size, variable path, and high variability |
| Bentriou et al., 2024 ^(45)^ | Frontiers in Oncology | Childhood Cancer | 7367 | Random survival forests (RSF), Cox proportional hazards with Lasso/Bootstrap Lasso | Dose-volume features, dosiomics features (whole heart and subparts) | late radio-induced high-grade cardiac disease | Prediction | RF model Dosiomics offers marginal improvement over traditional dose-volume metrics models; to estimate late RT-induced cardiac risk in childhood cancer survivors |
| Chen et al., 2024 ^(46)^ | Radiotherapy and Oncology | Lung cancer | 142 | Deep Learning (U-Net) | RT-Planning CT images, manual contours of 19 cardiac substructures (whole heart, chambers, great vessels, valves, coronary arteries) | Autosegmentation of 19 cardiac substructures (whole heart, chambers, great vessels, valves, coronary arteries) | Imaging/Segmentation | NN U-Net model accurately segments cardiac substructures, including coronary arteries, with high clinical acceptability; supports radiation dose studies and treatment planning |
| Yoo et al., 2024 ^(47)^ | Radiotherapy and Oncology | Lung cancer | 508 | LASSO for feature selection, Logistic regression; hybrid deep learning model, multilayer perceptron (MLP), convolutional neural network (CNN), recurrent network (RNN), vision transformer for prediction | 159 patient-specific features including clinical data, dosimetry (heart, sinoatrial node doses), diagnostic information | Radiation-induced atrial fibrillation (AF) | Prediction | Deep Learning-based models can predict radiation-induced AF in NSCLC patients, highlighting the importance of maximum heart and SAN dose in AF risk |
| Lai et al., 2024 ^(48)^ | Breast | Breast cancer | 1100 | Regression model | Individual left ventricle (LV) equivalent uniform dose (EUD), clinical risk factors, comorbidity status | Major adverse cardiac events (MACE) following radiotherapy | Prediction | Comorbid patients are more susceptible to cardiac events after breast RT; modified NTCP model provides individualized risk estimates incorporating dosimetric and clinical factors |
| Xie et al., 2024 ^(49)^ | Radiotherapy and Oncology | Lung cancer | 822 | Deep Learning (U-Net) | Coronary computed-tomography angiography (CCTA) parameters including fat attenuation index (FAI), coronary artery calcium (CAC) score, CAD-RADS classification, stenosis severity, computed-tomography fractional flow reserve (CT-FFR); treatment type (CHT vs CRT) | Major adverse cardiovascular events (MACEs); CCTA-based coronary artery impairment | Prediction | AI-assisted CCTA can identify cancer treatment-related coronary impairments and predict MACEs in lung cancer patients receiving CHT or CRT |
| Choi et al., 2024 ^(50)^ | JCO Clinical Cancer Informatics | Lung cancer | 209 | "Tree based TPOT AutoML  framework" | FDG-PET radiomics features | Cardiac uptake on FDG-PET using  radiomics (No myocardial uptake, diffuse myocardial uptake, focal myocardial uptake) | Imaging/Radiomics | "Radiomics features from standard  18F-FDG PET/CT scans can  accurately predict cardiac FDG  uptake |
| Patil et al, 2024 ^(51)^ | Journal of Radiotherapy in  Practice | Breast cancer | 53 | Regression model | RT-Planning CT: Maximum heart distance,  total heart volume within  the tangential field, total  contoured heart volume,  and planning target volume | "Mean radiation  (gy) heart dose" | Prediction/Dosiomics | Elastic Net models using standard  anatomical features can accurately  predict mean heart dose and  dose-volume metrics in left-sided  breast cancer patients undergoing  RT |
| Tohidinezhad et al., 2024 ^(52)^ | Radiotherapy and Oncology | Lung cancer | 374 | Regression model | Clinical factors (age, BMI, alcohol use, lung function, cardiac procedure history)  Tumor characteristics  Radiation dosimetry (Heart volume, LA Dmax)  Baseline EKG and lab data | New onset Atrial fibrillation (AF) | Prediction | A model that combines clinical and cardiac substructure dose can identify NSCLC patients at high-risk for post-radiotherapy atrial fibrillation |
| Kehayias et al., 2024 ^(53)^ | Frontiers in Oncology | Multiple cancers | Unspecified | Deep Learning | Planning CT images; clinician drawn contours | Autosegmentation of cardiovascular substructures, myocardial uptake | Imaging/Segmentation | Automated AI infrastructure can be deployed prospectively in routine RO workflows |
| Walls et al., 2024 ^(54)^ | Clinical Oncology | Lung cancer | 478 | Deep learning for auto-segmentation and regression model for prediction | Heart dose metrics (Dmax), patient demographics, tumor stage, treatment factors (PTV, lung dose), CVD risk factors, established CVD | Overall survival; incidence of symptomatic cardiac events (atrial fibrillation, HF, ACS) | Prediction/Dosiomics | RT dose to the heart base is independently associated with both mortality and clinically significant cardiac events in NSCLC |
| van der Pol et al., 2024 ^(55)^ | Physics and Imaging in Radiation Oncology | Lung cancer | 730 | Deep learning (U-Net) for autosegmentation and ML for survival modeling (elastic net cox regression, random forests) | Cardiac substructure dose metrics, DL-derived contours, a/b assumptions, patient demographics, tumor and treatment characteristics (PTV, pathology, age, sex) | Overall survival; Identification of influential cardiac substructure dose parameters, LA mean dose threshold | Imaging/Segmentation | DL identified the LA mean dose as the most influential cardiac substructure predictor of OS in lung SBRT patients |
| Loap et al., 2025 ^(56)^ | Strahlentherapie and Onkologie, Journal of Radiation Oncology, Biology, Physics | Breast cancer | 24 | Deep Learning | CT scans of breast cancer patients in the lateral decubitus (LDP) position | Accuracy and efficiency of automated organ-at-risk (OAR) and planning target volume (PTV) contouring | Imaging/Segmentation | Neural network can automatically contour OARs and PTVs for LDP breast RT with high accuracy and efficiency |
| Qiao ET AL., 2025 ^(57)^ | Frontiers in Cardiovascular Medicine | Breast and Lung | 984 | Extreme Gradient Boosting (XGBoost) | Clinical variables, RT dosimetry, CT-derived cardiac substructure segmentations, atrial volume indices (RAVI, LAVI) | Major adverse cardiac events (MACE) after thoracic RT | Prediction | A novel ML-identified predictor of MACE after thoracic RT and remains consistent across external lung and breast cancer cohorts |
| Lee et al., 2025 ^(58)^ | International Journal of Radiation Oncology Biology Physics | Hepatocellular carcinoma | 1893 | Logistic regression for prediction, deep learning for segmentation | 15 clinical variables (age, hypertension, smoking, history of cardiac disease, etc.) and 88 dosimetric parameters (i.e., heart V5) | Major adverse cardiac events (MACEs, grade ≥3 CTCAE v5.0); coronary artery disease as secondary outcome | Prediction | ML model accurately predicts MACEs in HCC patients receiving SBRT; useful for cardiac dose optimization in SBRT planning |
| Rajurkar et al., 2025 ^(59)^ | Journal of Applied Clinical Medical Physics | Breast cancer | 103 | Regression model | RT-planning CT, Haller Index, maximum heart distance (MHD), dosimetric parameters; mean heart dose (HMD) | Predicted mean heart dose under volunteer deep inspiration breath hold (vDIBH) | Prediction/Dosiomics | Supervised ML algorithm can predict cardiac dose before treatment planning, improving safety and efficacy of left breast RTwith vDIBH |
| Cruz-Chamorro et al., 2025 ^(60)^ | Radiotherapy and Oncology | Lung cancer | 155 | Regression model | Internal target volume (ITV)/lung volume ratio, chemoradiotherapy sequencing, tumor volume, involvement of pericardial lymph node stations (4R/L, 7-12), cardiac dose | Overall survival (OS) | Prediction/Dosiomics | Overall survival in post-op NSCLC is driven primarily by tumor-related factors (ITV/lung ratio, nodal involvement), not by cardiac dose. |
| Talebi et al., 2025 ^(61)^ | Journal of Applied Clinical Medical Physics | Breast cancer | 83 | Neural network (NN), generalized linear model (GLM), Naive Bayes (NB), support vector machine (SVM), and random forest (RF) | Cardiac dose-volume histograms, demographic data, echocardiographic parameters (2D/3D echo), radiomics features from ultrasound imaging | RT-induced cardiotoxicity at 6 months | Prediction | Integrating clinical and imaging features with dosimetric data substantially improves prediction of cardiotoxicity after breast cancer radiotherapy |
| Dincer et al., 2025 ^(62)^ | Frontiers in Oncology | Breast cancer | 42 | Gradient-boosted classification | Clinical data, heart-specific high-sensitivity Troponin-T (hs-TropT), radiomics features (from imaging), dosiomics features (heart-segmented dose descriptors) | Treatment-related cardiac events (subclinical cardiotoxicity) | Prediction | Integration of radiomics and dosiomics provides superior ML predictive capability for early cardiac events compared to traditional clinical and dosimetric parameters |
| Long et al., 2025 ^(63)^ | Academic Radiology | Thoracic Cancers | 125 | ML optimal biomarker (OBM), SVM | Pre-treatment radiomic features on CT from cardiovascular structures (including those based on texture, intensity, and morphology,  were extracted for a total of 1078 features per object.) | Development of cardiotoxicity | Prediction | Cardiothoracic toxicity can be feasibly predicted with high accuracy in patients with pre-treatment CT imaging via ML techniques |
| Cai et al., 2025 ^(64)^ | International Journal of Radiation Oncology, Biology, Physics | Esophageal cancer | 482 | Regression model | Changes in segmental myocardial 18F-fluorodeoxyglucose (FDG) uptake in PET/CT (mean and maximum standardized uptake values (SUVs)). Dose volume parameters (DVH) | Major adverse cardiac events (MACEs) grade ≥3, and symptomatic valvular disease | Prediction | Changes in basal myocardial FDG uptake are promising biomarkers for predicting radiation-induced cardiotoxicity. |
| Ansari et al., 2025 ^(65)^ | Journal of Medical Signals and Sensors | Breast cancer | 54 | Supervised machine-learning classifiers: Random Forest (RF), Decision Tree (DT), and K-Nearest Neighbors (KNN) using radiomic, dosimetric, and clinical features; deep learning (SwinUNETR, nnUNet) used only for heart autosegmentation | Demographics (e.g. age, BMI, comorbidities), dosimetric features, and radiomic features extracted from CT heart segmentation | Prediction of chemoradiation-induced heart failure (based on EF change at 3 years | Prediction | Combining radiomic, dosimetric, and clinical features with ML is feasible for predicting chemoradiation-induced heart failure in breast cancer patients |

1.	Wu J, Mazur TR, Ruan S, Lian C, Daniel N, Lashmett H, Ochoa L, Zoberi I, Anastasio MA, Gach HM, Mutic S, Thomas M, Li H. A deep Boltzmann machine-driven level set method for heart motion tracking using cine MRI images. Med Image Anal. 2018;47:68-80.

2.	Gernaat SAM, van Velzen SGM, Koh V, Emaus MJ, Isgum I, Lessmann N, Moes S, Jacobson A, Tan PW, Grobbee DE, van den Bongard DHJ, Tang JI, Verkooijen HM. Automatic quantification of calcifications in the coronary arteries and thoracic aorta on radiotherapy planning CT scans of Western and Asian breast cancer patients. Radiother Oncol. 2018;127(3):487-92.

3.	Emaus MJ, Isgum I, van Velzen SGM, van den Bongard H, Gernaat SAM, Lessmann N, Sattler MGA, Teske AJ, Penninkhof J, Meijer H, Pignol JP, Verkooijen HM, Bragatston study g. Bragatston study protocol: a multicentre cohort study on automated quantification of cardiovascular calcifications on radiotherapy planning CT scans for cardiovascular risk prediction in patients with breast cancer. BMJ Open. 2019;9(7):e028752.

4.	van Velzen SGM, Lessmann N, Velthuis BK, Bank IEM, van den Bongard D, Leiner T, de Jong PA, Veldhuis WB, Correa A, Terry JG, Carr JJ, Viergever MA, Verkooijen HM, Isgum I. Deep Learning for Automatic Calcium Scoring in CT: Validation Using Multiple Cardiac CT and Chest CT Protocols. Radiology. 2020;295(1):66-79.

5.	Niedzielski JS, Wei X, Xu T, Gomez DR, Liao Z, Bankson JA, Lai SY, Court LE, Yang J. Development and application of an elastic net logistic regression model to investigate the impact of cardiac substructure dose on radiation-induced pericardial effusion in patients with NSCLC. Acta Oncol. 2020;59(10):1193-200.

6.	Chan ST, Ruan D, Shaverdian N, Raghavan G, Cao M, Lee P. Effect of Radiation Doses to the Heart on Survival for Stereotactic Ablative Radiotherapy for Early-stage Non-Small-cell Lung Cancer: An Artificial Neural Network Approach. Clin Lung Cancer. 2020;21(2):136-44 e1.

7.	Loap P, Tkatchenko N, Kirova Y. Evaluation of a delineation software for cardiac atlas-based autosegmentation: An example of the use of artificial intelligence in modern radiotherapy. Cancer Radiother. 2020;24(8):826-33.

8.	Morris ED, Aldridge K, Ghanem AI, Zhu S, Glide-Hurst CK. Incorporating sensitive cardiac substructure sparing into radiation therapy planning. J Appl Clin Med Phys. 2020;21(11):195-204.

9.	Zhou Y, Hou Y, Hussain M, Brown SA, Budd T, Tang WHW, Abraham J, Xu B, Shah C, Moudgil R, Popovic Z, Cho L, Kanj M, Watson C, Griffin B, Chung MK, Kapadia S, Svensson L, Collier P, Cheng F. Machine Learning-Based Risk Assessment for Cancer Therapy-Related Cardiac Dysfunction in 4300 Longitudinal Oncology Patients. J Am Heart Assoc. 2020;9(23):e019628.

10.	Xu J, Wang J, Zhao F, Hu W, Yao G, Lu Z, Yan S. The benefits evaluation of abdominal deep inspiration breath hold based on knowledge-based radiotherapy treatment planning for left-sided breast cancer. J Appl Clin Med Phys. 2020;21(10):89-96.

11.	Gunturkun F, Akbilgic O, Davis RL, Armstrong GT, Howell RM, Jefferies JL, Ness KK, Karabayir I, Lucas JT, Jr., Srivastava DK, Hudson MM, Robison LL, Soliman EZ, Mulrooney DA. Artificial Intelligence-Assisted Prediction of Late-Onset Cardiomyopathy Among Childhood Cancer Survivors. JCO Clin Cancer Inform. 2021;5:459-68.

12.	Harms J, Lei Y, Tian S, McCall NS, Higgins KA, Bradley JD, Curran WJ, Liu T, Yang X. Automatic delineation of cardiac substructures using a region-based fully convolutional network. Med Phys. 2021;48(6):2867-76.

13.	Garrett Fernandes M, Bussink J, Stam B, Wijsman R, Schinagl DAX, Monshouwer R, Teuwen J. Deep learning model for automatic contouring of cardiovascular substructures on radiotherapy planning CT images: Dosimetric validation and reader study based clinical acceptability testing. Radiother Oncol. 2021;165:52-9.

14.	Zeleznik R, Weiss J, Taron J, Guthier C, Bitterman DS, Hancox C, Kann BH, Kim DW, Punglia RS, Bredfeldt J, Foldyna B, Eslami P, Lu MT, Hoffmann U, Mak R, Aerts H. Deep-learning system to improve the quality and efficiency of volumetric heart segmentation for breast cancer. NPJ Digit Med. 2021;4(1):43.

15.	Gal R, van Velzen SGM, Hooning MJ, Emaus MJ, van der Leij F, Gregorowitsch ML, Blezer ELA, Gernaat SAM, Lessmann N, Sattler MGA, Leiner T, de Jong PA, Teske AJ, Verloop J, Penninkhof JJ, Vaartjes I, Meijer H, van Tol-Geerdink JJ, Pignol JP, van den Bongard D, Isgum I, Verkooijen HM. Identification of Risk of Cardiovascular Disease by Automatic Quantification of Coronary Artery Calcifications on Radiotherapy Planning CT Scans in Patients With Breast Cancer. JAMA Oncol. 2021;7(7):1024-32.

16.	van Velzen SGM, Bruns S, Wolterink JM, Leiner T, Viergever MA, Verkooijen HM, Isgum I. AI-Based Quantification of Planned Radiation Therapy Dose to Cardiac Structures and Coronary Arteries in Patients With Breast Cancer. Int J Radiat Oncol Biol Phys. 2022;112(3):611-20.

17.	van Velzen SGM, Gal R, Teske AJ, van der Leij F, van den Bongard D, Viergever MA, Verkooijen HM, Isgum I. AI-Based Radiation Dose Quantification for Estimation of Heart Disease Risk in Breast Cancer Survivors After Radiation Therapy. Int J Radiat Oncol Biol Phys. 2022;112(3):621-32.

18.	Kim K, Chung SY, Oh C, Cho I, Kim KH, Byun HK, Yoon HI, Oh J, Chang JS. Automated coronary artery calcium scoring in patients with breast cancer to assess the risk of heart disease following adjuvant radiation therapy. Breast. 2022;65:77-83.

19.	van den Oever LB, Spoor DS, Crijns APG, Vliegenthart R, Oudkerk M, Veldhuis RNJ, de Bock GH, van Ooijen PMA. Automatic Cardiac Structure Contouring for Small Datasets with Cascaded Deep Learning Models. J Med Syst. 2022;46(5):22.

20.	Koide Y, Aoyama T, Shimizu H, Kitagawa T, Miyauchi R, Tachibana H, Kodaira T. Development of deep learning chest X-ray model for cardiac dose prediction in left-sided breast cancer radiotherapy. Sci Rep. 2022;12(1):13706.

21.	Bitterman DS, Selesnick P, Bredfeldt J, Williams CL, Guthier C, Huynh E, Kozono DE, Lewis JH, Cormack RA, Carpenter CM, Mak RH, Atkins KM. Dosimetric Planning Tradeoffs to Reduce Heart Dose Using Machine Learning-Guided Decision Support Software in Patients with Lung Cancer. Int J Radiat Oncol Biol Phys. 2022;112(4):996-1003.

22.	Atkins KM, Weiss J, Zeleznik R, Bitterman DS, Chaunzwa TL, Huynh E, Guthier C, Kozono DE, Lewis JH, Tamarappoo BK, Nohria A, Hoffmann U, Aerts H, Mak RH. Elevated Coronary Artery Calcium Quantified by a Validated Deep Learning Model From Lung Cancer Radiotherapy Planning Scans Predicts Mortality. JCO Clin Cancer Inform. 2022;6:e2100095.

23.	Momin S, Wolf J, Roper J, Lei Y, Liu T, Bradley JD, Higgins K, Yang X, Zhang J. Enhanced cardiac substructure sparing through knowledge-based treatment planning for non-small cell lung cancer radiotherapy. Front Oncol. 2022;12:1055428.

24.	Radici L, Ferrario S, Borca VC, Cante D, Paolini M, Piva C, Baratto L, Franco P, La Porta MR. Implementation of a Commercial Deep Learning-Based Auto Segmentation Software in Radiotherapy: Evaluation of Effectiveness and Impact on Workflow. Life (Basel). 2022;12(12).

25.	Momin S, Lei Y, McCall NS, Zhang J, Roper J, Harms J, Tian S, Lloyd MS, Liu T, Bradley JD, Higgins K, Yang X. Mutual enhancing learning-based automatic segmentation of CT cardiac substructure. Phys Med Biol. 2022;67(10).

26.	Smith AG, Petersen J, Terrones-Campos C, Berthelsen AK, Forbes NJ, Darkner S, Specht L, Vogelius IR. RootPainter3D: Interactive-machine-learning enables rapid and accurate contouring for radiotherapy. Med Phys. 2022;49(1):461-73.

27.	Chun J, Chang JS, Oh C, Park I, Choi MS, Hong CS, Kim H, Yang G, Moon JY, Chung SY, Suh YJ, Kim JS. Synthetic contrast-enhanced computed tomography generation using a deep convolutional neural network for cardiac substructure delineation in breast cancer radiation therapy: a feasibility study. Radiat Oncol. 2022;17(1):83.

28.	Walls GM, Giacometti V, Apte A, Thor M, McCann C, Hanna GG, O'Connor J, Deasy JO, Hounsell AR, Butterworth KT, Cole AJ, Jain S, McGarry CK. Validation of an established deep learning auto-segmentation tool for cardiac substructures in 4D radiotherapy planning scans. Phys Imaging Radiat Oncol. 2022;23:118-26.

29.	Choi BS, Yoo SK, Moon J, Chung SY, Oh J, Baek S, Kim Y, Chang JS, Kim H, Kim JS. Acute coronary event (ACE) prediction following breast radiotherapy by features extracted from 3D CT, dose, and cardiac structures. Med Phys. 2023;50(10):6409-20.

30.	Vendrame A CC, Chiovati P, Vinante L, Parvej M, Caroli A, Pirrone G, Barresi L, Drigo A, Avanzo M. Artificial Intelligence-Based Patient Selection for Deep Inspiration Breath-Hold Breast Radiotherapy from Respiratory Signals. Applied Sciences. 2023;13(8):4962.

31.	Walls GM, O'Connor J, Harbinson M, McCarron EP, Duane F, McCann C, McKavanagh P, Johnston DI, Erekkath J, Giacometti V, Gavin AT, McAleese J, Hounsell AR, Cole AJ, Butterworth KT, McGarry CK, Hanna GG, Jain S. Association between statin therapy dose intensity and radiation cardiotoxicity in non-small cell lung cancer: Results from the NI-HEART study. Radiother Oncol. 2023;186:109762.

32.	Haseltine JM, Apte A, Jackson A, Yorke E, Yu AF, Plodkowski A, Wu A, Peleg A, Al-Sadawi M, Iocolano M, Gelblum D, Shaverdian N, Simone CB, 2nd, Rimner A, Gomez DR, Shepherd AF, Thor M. Association of cardiac calcium burden with overall survival after radiotherapy for non-small cell lung cancer. Phys Imaging Radiat Oncol. 2023;25:100410.

33.	Chounta S, Allodji R, Vakalopoulou M, Bentriou M, Do DT, De Vathaire F, Diallo I, Fresneau B, Charrier T, Zossou V, Christodoulidis S, Lemler S, Letort Le Chevalier V. Dosiomics-Based Prediction of Radiation-Induced Valvulopathy after Childhood Cancer. Cancers (Basel). 2023;15(12).

34.	Ladbury C, Li RC, Danesharasteh A, Ertem Z, Tam A, Liu JS, Hao CR, Li RS, McGee H, Sampath S, Williams T, Glaser S, Khasawneh M, Liao ZX, Lee P, Ryckman J, Shaikh P, Amini A. Explainable Artificial Intelligence to Identify Dosimetric Predictors of Toxicity in Patients with Locally Advanced Non-Small Cell Lung Cancer: A Secondary Analysis of RTOG 0617. Int J Radiat Oncol. 2023;117(5):1287-96.

35.	Lee SH, Geng H, Arnold J, Caruana R, Fan Y, Rosen MA, Apte AP, Deasy JO, Bradley JD, Xiao Y. Interpretable Machine Learning for Choosing Radiation Dose-volume Constraints on Cardio-pulmonary Substructures Associated with Overall Survival in NRG Oncology RTOG 0617. Int J Radiat Oncol Biol Phys. 2023;117(5):1270-86.

36.	Finnegan RN, Chin V, Chlap P, Haidar A, Otton J, Dowling J, Thwaites DI, Vinod SK, Delaney GP, Holloway L. Open-source, fully-automated hybrid cardiac substructure segmentation: development and optimisation. Phys Eng Sci Med. 2023;46(1):377-93.

37.	Monlezun DJ. Percutaneous Coronary Intervention Mortality, Cost, Complications, and Disparities after Radiation Therapy: Artificial Intelligence-Augmented, Cost Effectiveness, and Computational Ethical Analysis. J Cardiovasc Dev Dis. 2023;10(11).

38.	Jemal A, Simard EP, Dorell C, Noone AM, Markowitz LE, Kohler B, Eheman C, Saraiya M, Bandi P, Saslow D, Cronin KA, Watson M, Schiffman M, Henley SJ, Schymura MJ, Anderson RN, Yankey D, Edwards BK. Annual Report to the Nation on the Status of Cancer, 1975-2009, featuring the burden and trends in human papillomavirus(HPV)-associated cancers and HPV vaccination coverage levels. J Natl Cancer Inst. 2013;105(3):175-201.

39.	Chin V, Finnegan RN, Chlap P, Otton J, Haidar A, Holloway L, Thwaites DI, Dowling J, Delaney GP, Vinod SK. Validation of a Fully Automated Hybrid Deep Learning Cardiac Substructure Segmentation Tool for Contouring and Dose Evaluation in Lung Cancer Radiotherapy. Clin Oncol (R Coll Radiol). 2023;35(6):370-81.

40.	Bernasconi A, Zanga, A., Lucas, P.J.F., Scutari, M., Trama, A., Stella, F. A causal network model to estimate the cardiotoxic effect of oncological treatments in young breast cancer survivors. Progress in Artificial Intelligence. 2024.

41.	Borges MG, Gruenwaldt J, Barsanelli DM, Ishikawa KE, Stuart SR. Automatic segmentation of cardiac structures can change the way we evaluate dose limits for radiotherapy in the left breast. J Med Imaging Radiat Sci. 2025;56(2):101844.

42.	Chin V, Chlap P, Finnegan R, Hau E, Ong A, Ma X, Descallar J, Otton J, Holloway L, Delaney GP, Vinod SK. Cardiac Substructure Dose and Survival in Stereotactic Radiotherapy for Lung Cancer: Results of the Multi-Centre SSBROC Trial. Clin Oncol (R Coll Radiol). 2024;36(10):642-50.

43.	Thor M, Scott JM, Reiner AS, Hong L, Yu AF, Apte A, McCormick B, Perez-Andujar A, Lee CP, Cervino L, Harrison J, Liang X, Patel P, Dauer LT, Moskowitz CS, Bernstein JL. Cardiopulmonary Substructure Doses are Not Correlated With Cardiorespiratory Fitness Among Breast Cancer Survivors Treated With Contemporary Radiation Therapy. Adv Radiat Oncol. 2024;9(12):101613.

44.	Mohan AT, K., Sankhe, M., Kiron, G. Maniar, P. Kolekar, A., Kushe, S., Kharote, P., Singh, A., Patel, K., Gupta, A., Patel, D., Bardeskar, N., Patkar, D. Clinical Feasibility of Artificial Intelligence-Based Autosegmentation of the Left Anterior Descending Artery in Radiotherapy for Breast Cancer. Indian Journal of Medical and Paediatric Oncology. 2024.

45.	Bentriou M, Letort V, Chounta S, Fresneau B, Do D, Haddy N, Diallo I, Journy N, Zidane M, Charrier T, Aba N, Ducos C, Zossou VS, de Vathaire F, Allodji RS, Lemler S. Combining dosiomics and machine learning methods for predicting severe cardiac diseases in childhood cancer survivors: the French Childhood Cancer Survivor Study. Front Oncol. 2024;14:1241221.

46.	Chen X, Mumme RP, Corrigan KL, Mukai-Sasaki Y, Koutroumpakis E, Palaskas NL, Nguyen CM, Zhao Y, Huang K, Yu C, Xu T, Daniel A, Balter PA, Zhang X, Niedzielski JS, Shete SS, Deswal A, Court LE, Liao Z, Yang J. Deep learning-based automatic segmentation of cardiac substructures for lung cancers. Radiother Oncol. 2024;191:110061.

47.	Yoo SK, Kim KH, Noh JM, Oh J, Yang G, Kim J, Kim N, Kim H, Yoon HI. Development of learning-based predictive models for radiation-induced atrial fibrillation in non-small cell lung cancer patients by integrating patient-specific clinical, dosimetry, and diagnostic information. Radiother Oncol. 2024;201:110566.

48.	Lai TY, Hu YW, Wang TH, Chen JP, Shiau CY, Huang PI, Lai IC, Liu YM, Huang CC, Tseng LM, Huang N, Liu CJ. Estimating the risk of major adverse cardiac events following radiotherapy for left breast cancer using a modified generalized Lyman normal-tissue complication probability model. Breast. 2024;77:103788.

49.	Xie Y, Shen H, Xu Q, Tu C, Yang R, Liu T, Tang H, Miao Z, Zhang J. Evaluating coronary arteries and predicting MACEs using CCTA in lung cancer patients receiving chemotherapy or chemoradiotherapy. Radiother Oncol. 2024;200:110498.

50.	Choi W, Jia Y, Kwak J, Werner-Wasik M, Dicker AP, Simone NL, Storozynsky E, Jain V, Vinogradskiy Y. Novel Functional Radiomics for Prediction of Cardiac Positron Emission Tomography Avidity in Lung Cancer Radiotherapy. JCO Clin Cancer Inform. 2024;8:e2300241.

51.	Patil D ZM, Raj R, Devi S. . Predicting heart dose in left-sided breast cancer patients using volumetric modulated arc therapy: an anatomical feature-driven machine learning model. Journal of Radiotherapy in Practice. 2025;24:e13.

52.	Tohidinezhad F, Nurnberg L, Vaassen F, Ma Ter Bekke R, Jwl Aerts H, El Hendriks L, Dekker A, De Ruysscher D, Traverso A. Prediction of new-onset atrial fibrillation in patients with non-small cell lung cancer treated with curative-intent conventional radiotherapy. Radiother Oncol. 2024;201:110544.

53.	Kehayias CE, Yan Y, Bontempi D, Quirk S, Bitterman DS, Bredfeldt JS, Aerts H, Mak RH, Guthier CV. Prospective deployment of an automated implementation solution for artificial intelligence translation to clinical radiation oncology. Front Oncol. 2023;13:1305511.

54.	Walls GM, O'Connor J, Harbinson M, Duane F, McCann C, McKavanagh P, Johnston DI, Giacometti V, McAleese J, Hounsell AR, Cole AJ, Butterworth KT, McGarry CK, Hanna GG, Jain S. The Association of Incidental Radiation Dose to the Heart Base with Overall Survival and Cardiac Events after Curative-intent Radiotherapy for Non-small Cell Lung Cancer: Results from the NI-HEART Study. Clin Oncol (R Coll Radiol). 2024;36(2):119-27.

55.	van der Pol LHG, Pomp J, Mohamed Hoesein FAA, Raaymakers BW, Verhoeff JJC, Fast MF. The influence of cardiac substructure dose on survival in a large lung cancer stereotactic radiotherapy cohort using a robust personalized contour analysis. Phys Imaging Radiat Oncol. 2024;32:100686.

56.	Loap P, Monteil R, Kirova Y, Vu-Bezin J. Automated contouring for breast cancer radiotherapy in the isocentric lateral decubitus position: a neural network-based solution for enhanced precision and efficiency. Strahlenther Onkol. 2025;201(6):601-5.

57.	Qiao EM, He J, Silos KD, Gasho JO, Belen P, Bitterman DS, McKenzie E, Steers J, Guthier C, Nohria A, Lu MT, Aerts H, Nikolova AP, Mak RH, Atkins KM. Baseline atrial volume indices and major adverse cardiac events following thoracic radiotherapy. Front Cardiovasc Med. 2025;12:1560922.

58.	Lee HI, Son J, Cho B, Goh Y, Jung J, Park JH, Chie EK, Kim KS, Kim YH, Kang HC, Yoon SM. Development and Validation of a Prediction Model for Cardiac Events in Patients With Hepatocellular Carcinoma Undergoing Stereotactic Body Radiation Therapy. Int J Radiat Oncol Biol Phys. 2025;122(5):1272-81.

59.	Rajurkar SA, Verma T, Gupta R. Estimation of heart dose in left breast cancer radiotherapy: Assessment of vDIBH feasibility using the supervised machine learning algorithm. J Appl Clin Med Phys. 2025;26(2):e14595.

60.	Cruz-Chamorro R, Bryant JM, Henning J, Robinson LA, Lee DH, Fontaine J, Toloza E, Rosenberg SA, Kim J, Naqa IE, Dilling TJ. Factors impacting cardiac dose and overall survival in post-operative non-small cell lung cancer patients. Radiother Oncol. 2025;212:111117.

61.	Talebi A, Bitarafan-Rajabi A, Alizadeh-Asl A, Seilani P, Khajetash B, Hajianfar G, Tavakoli M. Machine learning based radiomics model to predict radiotherapy induced cardiotoxicity in breast cancer. J Appl Clin Med Phys. 2025;26(4):e14614.

62.	Dincer S, Akmansu M, Akyol O. Machine learning modeling of cancer treatment-related cardiac events in breast cancer: utilizing dosiomics and radiomics. Front Oncol. 2025;15:1557382.

63.	Long M, Alnoury M, Udupa JK, Tong Y, Wu C, Poole N, Mannikeri S, Ky B, Feigenberg SJ, Zou JW, O'Reilly S, Torigian DA. Prediction of Radiation Therapy Induced Cardiovascular Toxicity from Pretreatment CT Images in Patients with Thoracic Malignancy via an Optimal Biomarker Approach. Acad Radiol. 2025;32(4):1895-905.

64.	Cai G, Wang K, Zhao J, Huang B, Wang W, Wang X, Li C, Li J, Cheng B, Yu J, Meng X. Predictive Value of Changes in Basal Myocardial (18)F-Fluorodeoxyglucose Uptake for Cardiotoxicity in Patients With Locally Advanced Esophageal Cancer Receiving Definitive Radiation Therapy. Int J Radiat Oncol Biol Phys. 2025;121(3):626-39.

65.	Ansari F, Neshasteh-Riz A, Paydar R, Mohagheghi F, Felegari S, Beigi M, Cheraghi S. Radiomics Analysis on Computed Tomography Images for Prediction of Chemoradiation-induced Heart Failure in Breast Cancer by Machine Learning Models. J Med Signals Sens. 2025;15:14.
