# Supplementary material for: A systematic review of artificial intelligence in radiotherapy associated cardiovascular toxicity
Source: Cardiooncology. 2026 Jun 19;12:110. doi: 10.1186/s40959-026-00528-5 (PMC13543590; doi:10.1186/s40959-026-00528-5)
Supplement: Supplementary file 4 [file 40959_2026_528_MOESM4_ESM.zip › Supplementary Tables 2-4.pdf]

Supplementary Table 2: Performance Metrics, Median AUC Scores of different ML models across all predictive modeling studies

AUC

Column1	Regression models	Neural Networks	Boosting Models	Random Forest	Support Vector Machine	Naive Bayes	K-Nearest Neighbors
Minimum	0.66	0.694	0.66	0.66	0.66	0.73	0.66
25% Percentile	0.7788	0.74	0.7485	0.77	0.745	0.735	0.787
Median	0.8045	0.817	0.807	0.802	0.8045	0.8	0.807
75% Percentile	0.845	0.915	0.886	0.86	0.858	0.94	0.882
Maximum	0.97	0.98	0.96	0.98	0.97	0.97	0.98
Range	0.31	0.286	0.3	0.32	0.31	0.24	0.32

Supplementary Table 3: Performance Metrics, Median sensitivity scores of different ML models across all predictive modeling studies

Sensitivity

Column1	Regression models	Neural Networks	Boosting Models	Random Forest
Number of values	5	5	1	7
Minimum	0.66	0.801	0.78	0.59
25% Percentile	0.73	0.8155	0.78	0.62
Median	0.8167	0.8333	0.78	0.73
75% Percentile	0.8987	0.8452	0.78	0.78
Maximum	0.964	0.857	0.78	0.83
Range	0.304	0.056	0	0.24

Supplementary Table 4: Performance Metrics, Median specificity scores of different ML models across all predictive modeling studies

Specificity

Column1	Regression models	Neural Networks	Boosting Models	Random Forest
Number of values	5	5	1	7
Minimum	0.66	0.667	0.81	0.77
25% Percentile	0.6891	0.723	0.81	0.79
Median	0.7833	0.8	0.81	0.83
75% Percentile	0.855	0.8795	0.81	0.86
Maximum	0.86	0.909	0.81	0.88
Range	0.2	0.242	0	0.11
